# Replication stress caused by low MCM expression limits fetal erythropoiesis and hematopoietic stem cell functionality

**DOI:** 10.1038/ncomms9548

**Published:** 2015-10-12

**Authors:** Silvia Alvarez, Marcos Díaz, Johanna Flach, Sara Rodriguez-Acebes, Andrés J. López-Contreras, Dolores Martínez, Marta Cañamero, Oscar Fernández-Capetillo, Joan Isern, Emmanuelle Passegué, Juan Méndez

**Affiliations:** 1 DNA Replication Group, Spanish National Cancer Research Centre (CNIO), Melchor Fernández Almagro, 3, 28029 Madrid, Spain; 2 The Eli and Edythe Broad Center for Regeneration Medicine and Stem Cell Research, UCSF, San Francisco, 94143 California, USA; 3 Genomic Instability Group, Spanish National Cancer Research Centre (CNIO), Melchor Fernández Almagro, 3, 28029 Madrid, Spain; 4 Flow Cytometry Unit, Spanish National Cancer Research Centre (CNIO), Melchor Fernández Almagro, 3, 28029 Madrid, Spain; 5 Compared Pathology Unit, Spanish National Cancer Research Centre (CNIO), Melchor Fernández Almagro, 3, 28029 Madrid, Spain; 6 Spanish National Cardiovascular Research Center (CNIC), Melchor Fernández Almagro, 3, 28029 Madrid, Spain

## Abstract

Replicative stress during embryonic development influences ageing and predisposition to disease in adults. A protective mechanism against replicative stress is provided by the licensing of thousands of origins in G1 that are not necessarily activated in the subsequent S-phase. These ‘dormant’ origins provide a backup in the presence of stalled forks and may confer flexibility to the replication program in specific cell types during differentiation, a role that has remained unexplored. Here we show, using a mouse strain with hypomorphic expression of the origin licensing factor mini-chromosome maintenance (MCM)3 that limiting origin licensing *in vivo* affects the functionality of hematopoietic stem cells and the differentiation of rapidly-dividing erythrocyte precursors. Mcm3-deficient erythroblasts display aberrant DNA replication patterns and fail to complete maturation, causing lethal anemia. Our results indicate that hematopoietic progenitors are particularly sensitive to replication stress, and full origin licensing ensures their correct differentiation and functionality.

## Abstract (web summary)

What causes hematopoietic stem cell loss of functionality? Here, Alvarez *et al*. show that loss of origin licensing factor MCM3 induces replicative stress (RS), causing aberrant erythrocyte maturation, but mice strains with higher tolerance to RS can overcome this defect.

## Graphical abstract

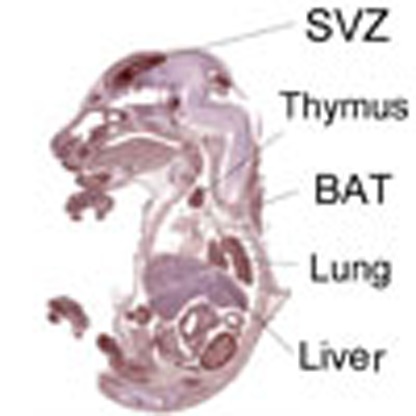

The process of genomic duplication starts at replication origins, which are licensed in the G1 phase of the cell division cycle, several hours before their activation in S phase. The licensing process is led by the origin recognition complex (ORC), cell division cycle 6 (CDC6) and Cdc10-dependent transcript 1 (CDT1) proteins, which cooperate to engage the mini-chromosome maintenance (MCM) complex with the DNA. MCM, composed by essential subunits MCM2-7, displays DNA helicase activity and becomes part of the replisome machinery (reviewed in references^1^,^2^). Defective control of DNA replication causes ‘replicative stress’ (RS), which is the underlying cause of several developmental diseases. Mutations in ORC, CDC6 and CDT1 genes are related to Meier-Gorlin syndrome, a type of dwarfism^3^,^4^,^5^, and mutations in MCM4 are linked to growth retardation, adrenal insufficiency and natural killer cell deficiency^6^. Impaired MCM function also increases cancer susceptibility^7^,^8^,^9^,^10^,^11^,^12^,^13^ (reviewed in references^14^,^15^).

MCM complexes are normally loaded onto DNA in excess relative to the number of origins that fire during the S phase (reviewed in reference^16^). One function of the surplus of MCM is to license dormant origins that may be activated in response to stalled or collapsed forks, providing a rescue mechanism under RS^17^,^18^,^19^. Another possible function for the high number of licensed origins, which remains largely underexplored, is to provide flexibility to the replication process during early embryonic development^20^,^21^ or in cell differentiation contexts that require the activation or shut-off of specific origins^22^,^23^,^24^ (reviewed in references^25^,^26^).

To investigate the protective effects of MCM against RS *in vivo*, a new Mcm3 conditional knockout (KO) mouse model was designed. The targeted allele, Mcm3-Lox, was hypomorphically expressed and caused embryonic lethality in homozygosity, which could be partially alleviated in a genetic background with enhanced resistance to RS. Our results show how a full complement of MCM proteins is specifically required to preserve the functionality of hematopoietic stem cells (HSCs) and the proper differentiation and maturation of erythrocytes in the developing embryo.

## Results

### A hypomorphic Mcm3 allele causes embryonic lethality

**Figure 1:**
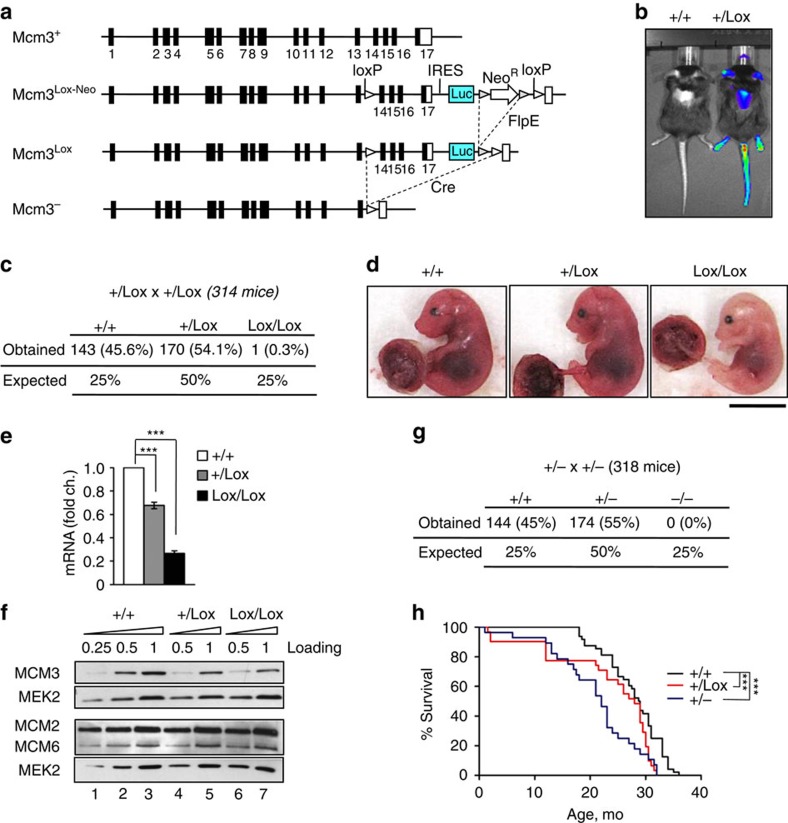
A mouse strain carrying a hypomorphic Mcm3 allele. (**a**) Schematic of the endogenous Mcm3 locus (Mcm3^+^) and the different alleles resulting from targeted recombination. Mcm3^Lox-Neo^ carries loxP sites flanking exons 14 and 17, a luciferase reporter expressed from an IRES element, and a neomycin-resistance cassette flanked by frt sites (gray triangles) that can be excised with FlpE recombinase, resulting in the Mcm3^Lox^ allele. Cre recombinase excises exons 14–17, resulting in the Mcm3^−^ (null) allele. (**b**) Imaging of whole body bioluminescence of Mcm3^+/+^ and Mcm3^+/Lox^ mice, taken 10 min after intraperitoneal injection of luciferin. Mice were partially shaved on the back to expose the skin. (**c**) Percentage (expected and obtained) of Mcm3^+/+^, Mcm3^+/Lox^ and Mcm3^Lox/Lox^ mice derived from extensive Mcm3^+/Lox^ × Mcm3^+/Lox^ breeding. (**d**) Representative images of Mcm3^+/+^, Mcm3^+/Lox^ and Mcm3^Lox/Lox^ E16.5 embryos and placental tissue. Scale bar, 1 cm. (**e**) Mcm3 mRNA levels in Mcm3^+/+^, Mcm3^+/Lox^ and Mcm3^Lox/Lox^ MEFs, determined by qRT-PCR. Histogram shows the average ±s.d. of 3 independent experiments. P-values were calculated with Fisher’s test (***P<0.001). (**f**) Protein levels of the indicated MCM subunits in Mcm3^+/+^, Mcm3^+/Lox^ and Mcm3^Lox/Lox^ MEFs as determined by immunoblots. For accurate comparisons, different amounts of each extract were loaded. For each SDS-PAGE, MEK2 levels are shown as loading control. Full western blots are shown in Supplementary Fig. 9A. (**g**) Percentage (expected and obtained) of Mcm3^+/+^, Mcm3^+/−^ and Mcm3^−/−^ mice after extensive Mcm3^+/−^ × Mcm3^+/−^ breeding. (**h**) Kaplan-Meier survival curves for Mcm3^+/+^ (black), Mcm3^+/Lox^ (red) and Mcm3^+/−^ (blue) mice. 42 individuals were included in the wt group (18 female; 24 male), 31 individuals in the +/Lox group (15 female; 16 male) and 35 individuals in the +/- group (13 female; 22 male). Log-Rank (Mantel-Cox) test indicated that there were significant differences between Mcm3^+/+^ and Mcm3^+/Lox^ or Mcm3^+/−^ curves (***P<0.001).

A modified mouse Mcm3 allele was designed with loxP sites flanking exons 14–17 and a luciferase reporter inserted at the 3′ UTR under the control of an IRES element. The resultant allele (Mcm3-Lox) was intended as a conditional KO, as Mcm3 expression could be ablated with Cre recombinase (Fig. 1a and Supplementary Fig. 1A,B). In addition, expression of Mcm3-Lox could be monitored by the bioluminescence activity associated to luciferase expression. Mcm3^+/Lox^ mice were born at Mendelian rates, and Mcm3-Lox expression in the skin and several internal organs was confirmed by luciferase activity after the administration of luciferin (Fig. 1b and Supplementary Fig. 1C). Unexpectedly, virtually no Mcm3^Lox/Lox^ individuals were born after extensive breeding of Mcm3^+/Lox^ mice (1 in 314; Fig. 1c). Mcm3^Lox/Lox^ embryos survived until E16.5-E18.5 but were noticeably smaller than wild-type (wt) or Mcm3^+/Lox^ littermates and their pale appearance suggested an anemic phenotype (Fig. 1d). Mcm3^+/Lox^ and Mcm3^Lox/Lox^ embryonic fibroblasts (MEFs) could be obtained and maintained in culture.

To determine the impact of the Mcm3-Lox modification on gene function, mRNA isolated from MCM3^Lox/Lox^ MEFs was sequenced to rule out the presence of any mutation that could affect MCM3 protein functionality (Supplementary Fig. 2). Next, Mcm3 expression was compared in MEFs derived from Mcm3^+/+^, Mcm3^+/Lox^ and Mcm3^Lox/Lox^ embryos. Mcm3 mRNA levels were reduced to approximately 70% in Mcm3^+/Lox^ and 30% in Mcm3^Lox/Lox^ MEFs, relative to the levels in Mcm3^+/+^ MEFs (Fig. 1e), and consistent reductions in MCM3 protein were observed in whole cell extracts (Fig. 1f; compare lanes 3–5–7). These results indicate that the Mcm3-Lox allele is hypomorphically expressed, presumably due to the modifications introduced in the 3′ UTR. This level of reduction in MCM3 did not significantly affect the cellular concentration of other MCM subunits (Fig. 1f).

At least in primary embryonic fibroblasts, downregulation of Mcm3 expression was compatible with DNA replication and cell proliferation (Supplementary Fig. 3A,B). Analyses of inter-origin distances (IOD) using stretched fibers revealed a similar frequency of origin activity between Mcm3^+/+^ and Mcm3^Lox/Lox^ MEFs in normal growth conditions (median IOD values of 118 and 116 Kb, respectively; Supplementary Fig. 3C). Upon challenging fork progression with aphidicolin, the IOD was shortened by 37% in Mcm3^+/+^ MEFs, as a consequence of the activation of dormant origins (median IOD 73.8 Kb; Supplementary Fig. 3C). In Mcm3^Lox/Lox^ MEFs, the reduction in IOD was limited to 24% (median IOD 87.5 Kb), reflecting a partial loss of back-up origins but also underscoring the large excess of origins licensed in G1, as even after the marked reduction in MCM3 protein concentration, a reservoir of additional origins were activated when needed. After several days in culture, Mcm3^Lox/Lox^ MEFs displayed γH2AX foci, a common marker of DNA damage associated to RS (Supplementary Fig. 3D).

### Mcm3-deficient mice are prone to haematological neoplasia

In contrast to Mcm3^Lox/Lox^, heterozygous Mcm3^+/Lox^ mice were viable and could be crossed with a CMV-Cre strain expressing Cre recombinase to generate viable Mcm3^+/−^ mice. As expected, breeding between Mcm3^+/−^ mice did not produce any Mcm3^−/−^ offspring (Fig. 1g). All previous attempts at making MCM-null models displayed pre-implantation lethality^8^,^9^. When cohorts of Mcm3^+/−^ and Mcm3^+/Lox^ mice were established for longevity studies, both groups presented a slight but significant reduction in lifespan (Fig. 1h) and an increased incidence of tumors, mainly lymphomas originated in the mesenteric lymph nodes that in multiple cases infiltrated to other organs. These tumors are not uncommon in aged mice but normally have a later onset and lower invasiveness (Supplementary Tables 1–3).

### DNA damage and incomplete erythropoiesis in the fetal liver

**Figure 2:**
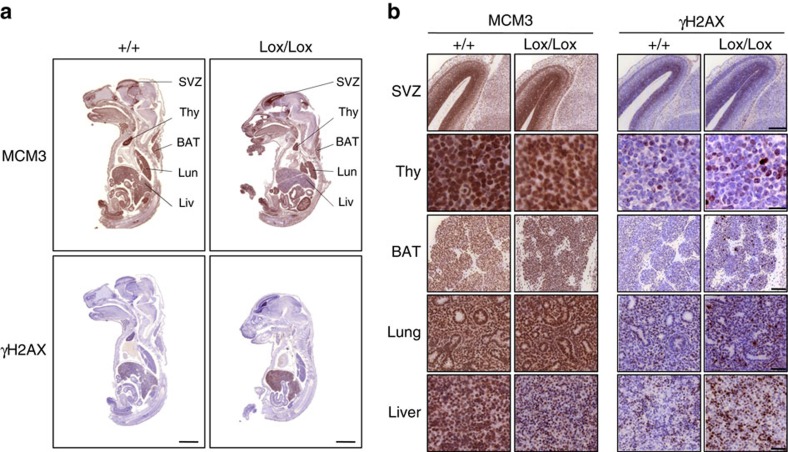
Hypomorphic Mcm3 expression and DNA damage in Mcm3^Lox/Lox^ embryos. (**a**) Immunohistochemistry (IHC) detection of MCM3 and γH2AX proteins in Mcm3^+/+^ and Mcm3^Lox/Lox^ embryo (E16.5) sections. The following tissues are indicated: SVZ, subventricular zone of the brain; Thy, thymus; BAT, brown adipose tissue; lung; liver. Scale bar, 2 mm. (**b**) Detailed sections of the indicated tissues stained for MCM3 and γH2AX. Scale bars: SVZ, 200 μm; thymus, 20 μm; BAT, 100 μm; lung, 50 μm; liver, 50 μm.

We next focused on the tissues that might be responsible for the lethality observed in Mcm3^Lox/Lox^ embryos, which by mid-late gestation (E14.5–18.5) were consistently smaller than their Mcm3^+/+^ or Mcm3^+/Lox^ counterparts. In control E16.5 embryos, MCM3 protein is readily detected by immunohistochemistry (IHC) in most tissues, particularly liver, lung, thymus, brown adipose tissue and the subventricular zone of the brain. In Mcm3^Lox/Lox^ embryos, the strong MCM3 staining in the liver was markedly reduced while γH2AX staining became apparent (Fig. 2). Mcm3 mRNA and protein levels confirmed the downregulation of Mcm3 expression in fetal liver extracts (Supplementary Fig. 4A,B). Global levels of cell proliferation were not affected, as monitored by Ki67 IHC staining (Supplementary Fig. 4C).

**Figure 3:**
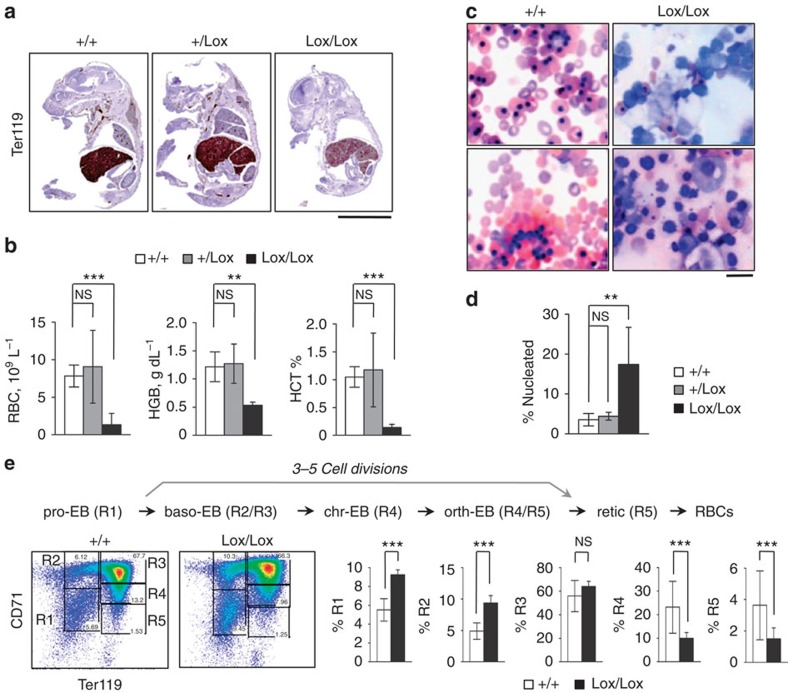
Defective erythrocyte maturation in Mcm3^Lox/Lox^ embryos. (**a**) Ter119 IHC staining in Mcm3^+/+^, Mcm3^+/Lox^ and Mcm3^Lox/Lox^ E16.5 embryos. Scale bar, 5 mm. (**b**) Peripheral blood counts from Mcm3^+/+^, Mcm3^+/Lox^ and Mcm3^Lox/Lox^ E14.5 embryos. RBC, number of red blood cells; HGB, haemoglobin concentration; HCT, hematocrit. Histograms show the average value±s.d. of Mcm3^+/+^ (*n*=6), Mcm3^+/Lox^ (*n*=7) and Mcm3^Lox/Lox^ (*n*=3) embryo peripheral blood cell counts. P-values were calculated by Fisher’s test (***P<0.001, **P<0.05, NS, not significant). (**c**) Representative fields of Giemsa-stained peripheral blood samples derived from Mcm3^+/+^ and Mcm3^Lox/Lox^ embryos. Scale bar, 20 μm. (**d**) Percentage of nucleated erythrocytes in embryo peripheral blood samples. Histograms show the average value±s.d. of Mcm3^+/+^ (*n*=5), Mcm3^+/Lox^ (*n*=8) and Mcm3^Lox/Lox^ (*n*=6) cell counts. Statistical significance was calculated by One-way Anova (**P<0.05, NS, not significant). (**e**) Top, schematic of the pathway of erythrocyte differentiation. Bottom left, density plots showing the distribution of EBs in R1-R5 stages in Mcm3^+/+^ and Mcm3^Lox/Lox^ fetal livers. Bottom right, histogram quantification of the percentage (±s.d.) of cells in R1-R5 in Mcm3^+/+^ (*n*=15) and Mcm3^Lox/Lox^ (*n*=7) fetal livers. P-values were calculated by Fisher’s test (***P<0.001, NS not significant).

The liver is the major site of fetal hematopoiesis from E11 until shortly before birth, when this process becomes established in the bone marrow. To evaluate the impact of Mcm3 downregulation in hematopoiesis, specific blood lineages were detected by IHC in mid-late gestation embryos. This approach did not reveal major differences in the abundance of mature B-lymphocytes (stained with Pax-5) and megakaryocytes (stained with FVIII) in the fetal liver, nor T-lymphocytes (stained with CD3) in the fetal thymus of Mcm3^Lox/Lox^ embryos compared to their Mcm3^+/+^ or Mcm3^+/Lox^ counterparts (Supplementary Fig. 5). In contrast, the staining with erythrocyte marker Ter119 was much reduced in the liver of Mcm3^Lox/Lox^ embryos (Fig. 3a). Peripheral blood from Mcm3-deficient E14.5 embryos displayed a marked decrease in red blood cells (RBCs), haemoglobin concentration and hematocrit (Fig. 3b), all consistent with a phenotype of fetal anemia. Giemsa staining of peripheral blood cells revealed a large accumulation of immature erythroblasts (EBs) with uncompacted nuclei, as well as the presence of micronuclei that are indicative of genomic instability (Fig. 3c,d).

Erythrocyte differentiation is coupled to 3–5 rounds of DNA replication and cell division^27^. Based on the expression of surface markers CD71 and Ter119, EBs can be separated by flow cytometry in consecutive differentiation stages^28^: pro-EBs (located in ‘region’ R1); early basophilic EBs (baso-EBs; R2); early and late baso-EBs (R3); chromatophilic/orthochromatophilic EBs (chr-EBs/ orth-EBs; R4); and late orth-EBs/ reticulocytes (R5). In agreement with the analyses of embryo peripheral blood, the fetal liver of Mcm3^Lox/Lox^ embryos displayed an accumulation of pro-EBs and early baso-EBs, and a concomitant reduction in chr-EBs, orth-EBs and mature reticulocytes (Fig. 3e). Activated caspase 3 was detected in a fraction of R3 Mcm3-deficient EBs, indicating apoptotic events (Supplementary Fig. 6).

### Impaired erythrocyte maturation upon transplantation

**Figure 4:**
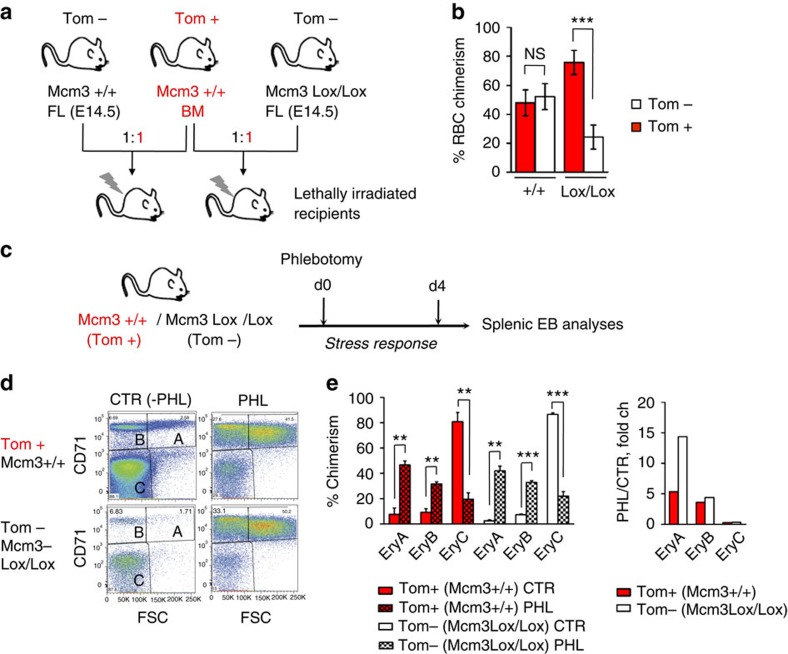
Impaired RBC maturation during steady-state or stress erythropoiesis. (**a**) Experimental design of a competitive transplantation assay between Mcm3^+/+^ and Mcm3^Lox/Lox^ fetal liver (FL; E14.5) cells and bone marrow (BM) cells of a tool strain constitutively expressing red fluorescent Tomato (Tom) protein, with wild-type Mcm3 levels. In each case, cells were transplanted at 1:1 ratio. (**b**) Quantification of the RBC (Ter119-positive) chimerism in peripheral blood of recipient mice, 2 months post-transplantation. Histograms show average±s.d.; *n*=4 mice transplanted with Mcm3^+/+^ cells and *n*=4 mice transplanted with Mcm3^Lox/Lox^ cells. P-values were calculated by Fisher’s test (***P<0.001, NS, not significant). (**c**) Schematic of a phlebotomy assay in chimaeric mice to monitor the erythropoietic stress response. (**d**) Examples of flow cytometry analyses of splenic EryA, EryB, EryC precursors (abbreviated A, B, C), in control mice (CTR) or mice subjected to phlebotomy (PHL). Analyses of EryA, EryB and EryC was carried out four days after the bleeding, differentiating between Tom+ and Tom- cells. (**e**) Left, histograms showing the percentage of EryA, EryB and EryC populations relative to control mice (*n*= 2 CTR, *n*=4 PHL mice). P-values were calculated by Fisher’s test (***P<0.001, **P<0.01). Right, histogram showing the fold-change variation in Tom+ (red) or Tom- (white) EryA, EryB and EryC populations induced by phlebotomy.

The fetal liver contains HSCs capable of reconstituting the entire hematopoietic system. In order to compare the maturation ability of wild-type and Mcm3-deficient cells towards the erythroid lineage, fetal liver cells (E14.5) derived from wild-type or Mcm3-deficient embryos were transplanted into lethally-irradiated recipient mice, in competition (1:1 ratio) with bone marrow cells that constitutively express a Tomato (Tom) fluorescent marker^29^ (Fig. 4a). Two months after transplantation, the contribution of control Mcm3^+/+^ cells (Tom-negative) towards mature RBCs in peripheral blood was approximately 50% in the chimaeras (Fig. 4b). In contrast, the contribution of Mcm3-deficient cells (Tom-negative) towards RBCs was much reduced, with Mcm3-proficient cells (Tom-positive) taking over most of the RBC population (Fig. 4b). These results confirm that EBs with reduced expression of Mcm3 do not mature properly under steady-state conditions.

Chimaeric Mcm3^+/+^ (Tom+) / Mcm3^Lox/Lox^ (Tom-) mice were also used to monitor the acute response to erythropoietic stress induced by phlebotomy (Fig. 4c). In this setting, cells in the spleen with Ter119 high content can be analyzed using the forward scatter parameter (FSC) and CD71 to resolve three EB subpopulations previously labelled as EryA, EryB and EryC^30^. EryA (Ter119^high^ CD71^high^ FSC^high^) correspond to baso-EBs; EryB (Ter119^high^ CD71^high^ FSC^low^) correspond to late baso-EBs and polychromatic-EBs; EryC (Ter119^high^ CD71^low^ FSC^low^) are orth-EBs and reticulocytes. In response to stress, the EryA and EryB subpopulations are rapidly increased^30^. Both Mcm3-competent and Mcm3-deficient EBs reacted to stress, as indicated by the accumulation of splenic EryA and EryB progenitors (Fig. 4d,e). However, the accumulation of EryA in Mcm3-deficient cells was almost 3-fold higher than in Mcm3^+/+^ cells, suggesting that the rapid RBC maturation required under stress conditions is also delayed or impaired (Fig. 4e, right).

### Aberrant DNA replication during erythrocyte maturation

**Figure 5:**
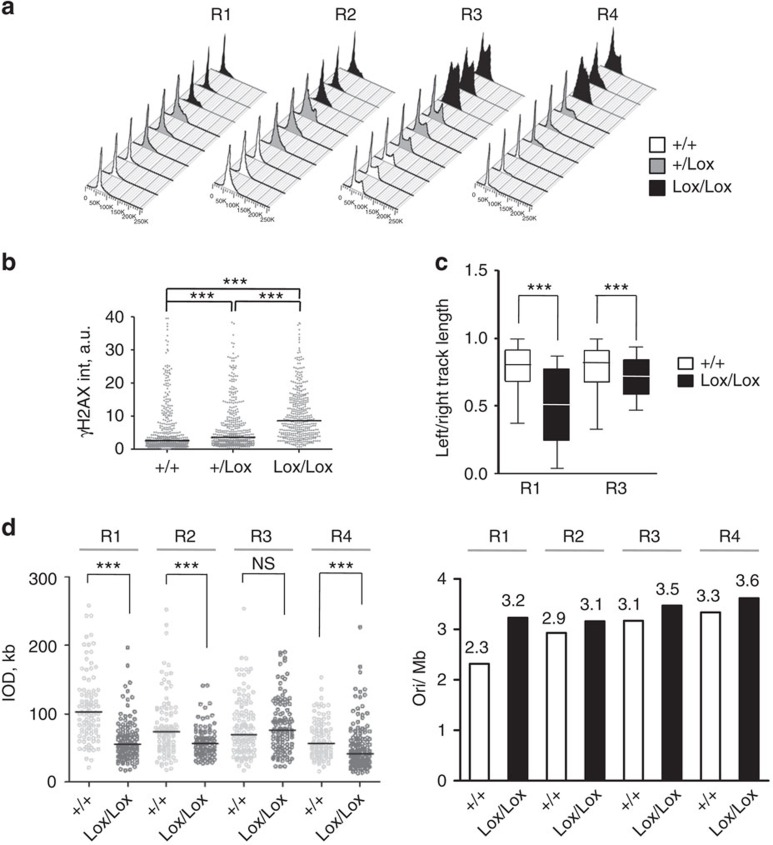
Aberrant DNA replication patterns in differentiating EBs. (**a**) DNA content of EBs in R1-R4 differentiation stages. Three examples of each genotype are shown. (**b**) γH2AX intensity in Mcm3^+/+^, Mcm3^+/Lox^ and Mcm3^Lox/Lox^ R3 EBs (>300 nuclei scored per condition). Data are representative of 3 independent experiments. P-values were calculated by Mann-Whitney test (***P<0.001). (**c**) Box plots showing the values of fork asymmetry in Mcm3^+/+^ and Mcm3^Lox/Lox^ EBs sorted in R1 and R3 stages. >180 bidirectional forks were counted per condition. Statistical analysis was done with Mann-Whitney rank sum test (***P<0.001). (**d**) Left, IOD values (data distribution and median value) of Mcm3^+/+^, Mcm3^+/Lox^ and Mcm3^Lox/Lox^ EBs at the indicated (R1-R4) maturation stage. >100 IOD values were scored for each condition. P-values were calculated using Mann-Whitney test (***P<0.001; NS, not significant). Right, origin density expressed by number of origins/Mb of DNA was estimated as indicated in Materials and Methods. See also Table 1.

To monitor whether inefficient erythrocyte maturation was related to DNA replication and cell cycle progression, fetal liver cells obtained from Mcm3^+/+^, Mcm3^+/Lox^ and Mcm3^Lox/Lox^ embryos were sorted at the R1-R4 stages (R5 was not included as most cells have started the enucleation process). Interestingly, Mcm3^Lox/Lox^ baso-EBs (R3) and chr-EBs (R4) displayed a prominent accumulation in S phase, indicative of problems to complete DNA replication (Fig. 5a). High levels of γH2AX and a higher frequency of fork asymmetry (indicative of stalled forks) were detected in Mcm3^Lox/Lox^ early EBs, consistent with RS interfering with RBC maturation (Fig. 5b,c).

Next, we analyzed the patterns of origin activity in EB populations sorted from embryonic livers using stretched DNA fibers labelled with thymidine analogs chloro-deoxyuridine (CldU) and iodo-deoxyuridine (IdU). These analyses revealed a dynamic pattern in which more origins are activated as EBs progress in the differentiation pathway. The median IOD was progressively reduced from approximately 100 Kb in pro-EB (R1) to 74 Kb in early baso-EBs (R2), 69 Kb in late baso-EBs (R3) and 56 Kb in orth-EBs (R4; Fig. 5d, left). The length of DNA fibers was similar in all populations, ruling out indirect effects on IOD measurements (Supplementary Fig. 7). Furthermore, the tendency towards more origin activity was confirmed by measuring origin density (Fig. 5d, right and Table 1). Interestingly, the patterns of origin activity were altered in Mcm3-deficient EBs, which displayed lower IOD values and slightly higher origin density (Fig. 5d and Table 1). As discussed below, this behaviour likely reflects the increased pressure on early EBs to replicate as a compensatory mechanism for the lack of mature RBCs.

### Mcm3^Lox/Lox^ lethality can be rescued by CHK1 overexpression

Because our results strongly point to RS as the underlying cause for incomplete RBC maturation, we considered the possibility that the embryonic lethality of the Mcm3^Lox/Lox^ genotype could be alleviated in a background with higher tolerance to RS, such as the recently described strain carrying an extra copy of checkpoint kinase CHK1 (ref. 31). Mcm3^+/Lox^:Chk1^+/Tg^ mice were generated by direct intercrossing between both strains, and further breading of Mcm3^+/Lox^:Chk1^+/Tg^ mice resulted in Mcm3^Lox/Lox^ individuals that successfully completed gestation, despite having low expression levels of Mcm3 (Supplementary Fig. 8). A few Mcm3^Lox/Lox^ individuals were born without the extra Chk1 allele, indicating that the change in genetic background (now mixed C57BL/6-CD1) also contributed to survival of the Mcm3^Lox/Lox^ embryos. Independently of the effect of the mixed background, the birth rate was biased towards Mcm3^Lox/Lox^: Chk1^+/Tg^ progeny. This partial rescue of embryonic lethality provides further genetic evidence that RS caused by Mcm3 downregulation is the reason for the phenotypes observed in the Mcm3^Lox/Lox^ strain.

### Impaired fitness of Mcm3^Lox/Lox^ hematopoietic stem cells

**Figure 6:**
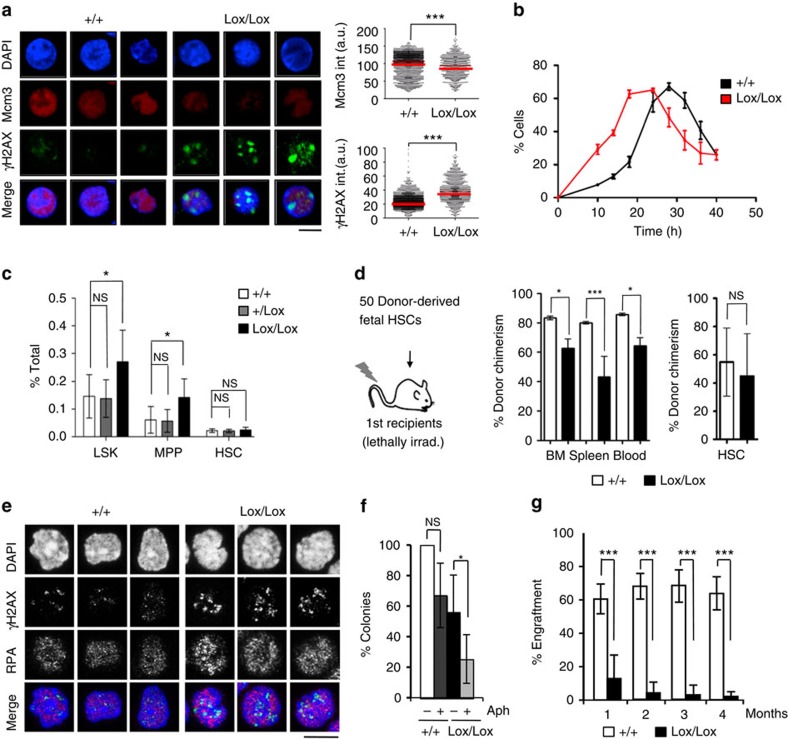
Impaired reconstitution potential of Mcm3-deficient HSCs. (**a**) Representative images of LSK cells (Lin^−^, cKit^+^, Sca1^+^) isolated from Mcm3^+/+^ and Mcm3^Lox/Lox^ embryonic liver, stained with MCM3 (red) and γH2AX (green) antibodies. Scale bar, 5 μM. Right panels, quantification of MCM3 or γH2AX staining intensity, using Definiens software on confocal microscopy images. Red lines indicate the median nuclear intensity (>800 nuclei scored per condition). P-values were calculated by Mann-Whitney test (***P<0.001). (**b**) Single-cell tracking of the first cell division of cultured fetal Mcm3^+/+^ or Mcm3^Lox/Lox^ HSCs. Representation shows the average±s.d. of 4 independent experiments. (**c**) Histograms showing the percentage of LSK, MPP and HSCs in Mcm3^+/+^, Mcm3^+/Lox^ and Mcm3^Lox/Lox^ embryonic liver tissue. Median values±s.d. are shown (*n*=7 embryos for each genotype). P-values were calculated by Fisher’s test (*P<0.05; NS, not significant). (**d**) HSC engraftment results at 4 months post-transplantation. Middle histograms show the percentage of donor-derived chimerism in the bone marrow (BM), spleen and peripheral blood. Right histograms show the percentage of chimerism in the adult HSC compartment. Quantification shows average±s.d. of three independent experiments. P-values were calculated by Fisher’s test (***P<0.001; *P<0.05; NS, not significant). (**e**) Confocal microscopy images of γH2AX and RPA staining in donor-derived, cycling adult Mcm3^+/+^ or Mcm3^Lox/Lox^ HSCs isolated from the BM. Scale bar, 10 μm. (**f**) Histogram showing the average number of colonies (±s.d.) formed 7 days after plating Mcm3^+/+^ or Mcm3^Lox/Lox^ HSCs in methylcellulose, in the absence or presence of low-dose aphidicolin (50 ng ml^−1^). Results are normalized for vehicle-treated cells (DMSO) and expressed as fold change compared to Mcm3^+/+^ HSCs. 3 independent experiments were conducted and included in the quantification. P-values were calculated using Fisher’s test (*P<0.05; NS, not significant). (**g**) Donor-derived adult Mcm3^+/+^ and Mcm3^Lox/Lox^ HSCs were re-isolated and transplanted into lethally irradiated secondary recipients (500 HSCs per mouse; 5 mice per group). Histogram shows the percentage of donor-derived chimerism in peripheral blood, assessed 1–4 months post-transplantation. Data are mean values±s.d. P-values were calculated using Fisher’s test (***P<0.001).

A recent report has linked RS to the limited functionality observed in aged HSCs^32^. Our Mcm3-deficient model provided a proper genetic tool to validate this concept, as Mcm3-deficient fetal HSCs should also be affected. Fetal Mcm3^Lox/Lox^ LSK cells (Lin^−^/cKit^+^/Sca1^+^), which comprise both HSCs (Lin^−^/cKit^+^/Sca1^+^/Flk2^−^/CD48^−^/CD150^+^) and multipotent progenitor cells (MPPs: Lin^−^/cKit^+^/Sca1^+^/Flk2^+^), had lower amounts of MCM3 protein and higher levels of RS marker γH2AX (Fig. 6a). Intriguingly, single-cell tracking of cell division revealed slightly faster proliferation kinetics of Mcm3^Lox/Lox^ than Mcm3^+/+^ fetal HSCs (Fig. 6b). This could reflect a pressure to proliferate and differentiate into MPPs, as it has been described for adult HSCs in the presence of DNA damage^33^. Consisting with this notion, immunophenotyping analyses revealed a two-fold increase in the concentration of LSK cells in Mcm3^Lox/Lox^ embryos, caused by the accumulation of MPPs (Fig. 6c).

To directly test their functionality, fetal HSCs were isolated from E14.5-E16.5 CD45.2 C57BL/6 Mcm3^+/+^, Mcm3^+/Lox^ and Mcm3^Lox/Lox^ embryos and transplanted into lethally irradiated congenic CD45.1 C57BL/6 recipient mice. Four months after transplantation, recipient mice displayed decreased Mcm3^Lox/Lox^ chimerism in all hematopoietic organs (Fig. 6d). Because formation of an adult HSC compartment was observed (Fig. 6d, right), donor-derived HSCs were re-isolated from the bone marrow of recipient mice to evaluate their proliferation potential *in vitro* and *in vivo*. Mcm3-deficient adult HSCs displayed RS markers γH2AX and RPA (Fig. 6e) and inefficient colony formation in methylcellulose in the presence of aphidicolin (Fig. 6f). Furthermore, when subjected to a major replication challenge such as a secondary transplantation, Mcm3^Lox/Lox^ adult HSCs were drastically affected in their ability to self-renew and regenerate the blood system (Fig. 6g).

## Discussion

Mcm2-7 are essential genes that encode key components of the main DNA helicase involved in genome replication. Mcm2-7 are highly expressed in proliferating cells, and DNA replication can still occur in cell lines after a significant reduction (>90% in some cases) in the cellular concentration of MCM protein complexes^17^,^18^,^19^. This tolerance to MCM downregulation is related to the fact that MCM complexes are engaged with DNA in large excess relative to the number of replication forks normally established during S phase. The surplus of MCM complexes license dormant origins that are activated only as a rescue mechanism when DNA replication is disrupted (reviewed in reference^34^). The use of dormant origins *in vivo* is well documented in the mouse, and MCM downregulation beyond ∼2/3 of its physiological levels causes embryonic lethality or promotes tumorigenesis in adults^7^,^8^,^9^,^10^,^11^,^12^,^13^. Therefore, it is likely that certain cell types in the developing embryo, such as stem and progenitor cells, are particularly sensitive to RS induced by low MCM levels.

We have tested the hypothesis that different cell types may have different requirements for MCM concentration using a novel strain with hypomorphic Mcm3 expression. While Mcm3^Lox/Lox^ MEFs proliferated and replicated DNA with approximately 1/3 of the normal concentration of MCM3 protein, a similar reduction severely impaired hematopoietic progenitors, indicating a stricter requirement for origin licensing in the latter. In mid-gestation, hematopoiesis in the fetal liver is largely geared towards the production of RBCs to guarantee oxygen delivery to the rapidly growing embryo. Interestingly, erythroid precursors undergo several rounds of DNA replication and cell division during terminal differentiation, and genetic models that ablate cell cycle regulators such as Rb, E2F4, E2F8 or D-cyclins frequently result in embryonic anemia^35^,^36^,^37^,^38^. While D-Cyclin/CDK and Rb/E2F constitute the axis of a large transcriptional pathway regulating multiple genes, here we report for the first time that downregulation of a single MCM gene is sufficient to impair hematopoietic progenitor cells, causing anemia. Cytological analyses of embryonic blood revealed lower counts of RBCs and abundance of immature nucleated erythroblasts. Furthermore, transplantation of Mcm3-deficient fetal liver cells into lethally irradiated mice reconstituted the adult RBC population with much lower efficiency than Mcm3-competent cells. Our results indicate that a full complement of MCM is required for erythrocyte maturation in steady-state conditions and in response to erythropoietic stress. If MCM levels drop to approximately 1/3 of its normal concentration, the severity of the anemia causes embryonic lethality in the C57BL/6 genetic background. As reported in other mouse models such as the Mcm4-chaos mutant^8^,^11^,^12^ or the Rif1 KO mouse^39^, Mcm3^Lox/Lox^ embryonic lethality was partially alleviated in a mixed C57BL/6-CD1 background and it was further rescued by overexpression of CHK1 kinase, reinforcing the connection between RS and the phenotypes observed.

To our knowledge, the single-molecule analyses of DNA replication in EB precursors isolated from the fetal liver provide the first evidence that the program of DNA replication undergoes active changes during the physiological maturation of mammalian erythrocytes. As pro-EBs proliferate and differentiate into mature reticulocytes, their replication program requires the progressive activation of more origins. This observation has interesting antecedents: chicken erythrocytic progenitors forced to differentiate *ex vivo* display a broadening in origin usage in the β-globin locus^23^. Also, origin firing is enhanced in murine erythroleukemia cells derived from transformed pro-EBs and forced to differentiate in culture^40^. It is conceivable that rapid DNA replication, driven from an increasingly high number of origins, is related to the rapid loss of DNA methylation marks observed during mouse erythropoiesis^41^. These patterns of DNA replication were altered in Mcm3-deficient pro-EBs, which displayed a higher frequency of origin activation and asymmetric forks from the start of their differentiation program. Later in differentiation, baso-EBs and chr-EBs were not capable of completing DNA replication, accumulated in S phase and activated the apoptotic program, thus preventing erythrocyte maturation. This likely triggers a compensation mechanism that pressures pro-EBs to replicate and proliferate, contributing to the aberrant replication patterns. Fork asymmetry reflects improper coordination between fork pairs^42^ and may also trigger the activation of compensatory origins. The fact that pro-EBs were capable of activating extra origins after Mcm3 downregulation underscores the fact that a very large excess of MCM proteins associate with DNA during G1.

Mid gestation is also the time of HSCs expansion in the fetal liver. At this stage, HSCs divide very rapidly (reviewed in reference^43^), which could also make them vulnerable to RS. The capacity of Mcm3^Lox/Lox^ fetal HSCs to reconstitute the immune system upon transplantation into irradiated recipients was compromised but not completely impaired, and a population of donor-derived adult HSCs was generated in recipient mice. The lack of engraftment potential was strikingly exacerbated when Mcm3^Lox/Lox^ HSCs were re-isolated from the bone marrow of recipient mice and tested in secondary transplantations. These observations are in line with the emerging concept that RS affects HSC functionality in older individuals. With age, HSCs lose reconstitution potential for causes that have been debated^44^,^45^,^46^,^47^,^48^ (reviewed in references^49^,^50^). Interestingly, a comparison of gene expression profiles of young versus old adult HSCs revealed that Mcm2-7 are downregulated in old HSCs, suggesting a new mechanistic link between faulty DNA replication and functional impairment^32^. The work presented here with a new mouse model with hypomorphic MCM expression provides direct genetic evidence that RS is a driving force behind HSC loss of functionality.

In summary, Mcm3 downregulation during embryonic development caused RS in hematopoietic progenitors leading to fetal anemia; in adults, it reduced life expectancy and promoted lymphomagenesis. Thus, future therapies designed to modulate RS are a promising way to fight ageing and hematopoietic malignancies.

## Methods

### Generation of Mcm3^+/Lox^ mouse strain

A targeting vector was designed that included an IRES-EGFP-luciferase reporter cassette after the Mcm3 stop codon located at exon 17, two loxP sites flanking Mcm3 exons 14 to 17, and a FRT-flanked neomycin-resistance cassette for selection. The linearised vector was electroporated into 129Sv/Pas ES cells, and its genomic integration by recombination was screened by PCR and Southern blotting. Verified transgenic ES clones (Mcm3-Lox-Neo) were microinjected into C57BL/6J blastocysts and germline transmission was confirmed. Vector preparation and genetic manipulations were conducted at Genoway (Lyon, France). Mice carrying the Mcm3-Lox-Neo allele were crossbred with a strain expressing Flp recombinase to eliminate the neomycin-resistance cassette, generating the Mcm3-Lox allele. Mcm3^+/Lox^ mice were crossbred with the CMV-Cre strain to generate the Mcm3-null allele, referred to as Mcm3^−^. Mice of both sexes were used in experiments, except when noted otherwise. Primers used for PCR genotyping are indicated in Supplementary Table 4.

### Mouse handling and luciferase detection

Mice were hosted at the CNIO animal facility, except for a cohort of Mcm3^+/Lox^ mice that were transferred to the UCSF animal facility for the analyses of HSCs. Animal procedures were approved by the Ethical Committee of the *Instituto de Salud Carlos III* (Madrid, Spain), and HSC transplantation experiments were performed under UCSF IACUC-approved protocols. For each experiment, sample size was calculated using Resource Equation^51^. *In vivo* luciferase imaging was performed in an IVIS imaging system (Caliper Life Sciences, CA) in mice anesthetized with 2.5% isoflurane, 10 min after an intraperitoneal injection of luciferin (150 mg kg^−1^ of body weight). Mice were partially shaved to monitor bioluminescence in the back skin. When indicated, mice were sacrificed 10 min after luciferin injection and necropsies were performed in order to monitor luciferase activity in internal organs.

### Mouse embryonic fibroblasts (MEFs) isolation and culture

Mcm3^+/+^, Mcm3^+/Lox^ and Mcm3^Lox/Lox^ primary MEFs were derived from E12.5–14.5 embryos resulting from crosses between Mcm3^+/Lox^ mice and cultured in Dulbecco’s modified minimal Eagle medium (DMEM) supplemented with 10% FBS and antibiotics. For cell proliferation curves, aliquots of 0.5 × 10^5^ cells were seeded and counted every 2 days in a hemocytometer. When indicated, 10 μM bromo-deoxyuridine (BrdU; Sigma) was added to the medium for 30 min before cell harvesting. Cells were fixed in 70% ethanol, washed in PBS and stained with 50 μg ml^−1^ propidium iodide (Sigma) in the presence of 10 μg ml^−1^ RNase A (Qiagen). Fixed cells were treated with 2 M HCl for 20 min and incubated with FITC-conjugated anti-BrdU antibody for 60 min. Flow cytometry data was acquired in a FACS Canto II (BD, San Jose, CA) and analyzed with FlowJo 9.7.5 (Tree Star, Ashland, OR).

### Quantitative RT–PCR, cell extracts and immunoblots

Total RNA was extracted using the RNeasy Mini Kit (Qiagen). 1 μg of total RNA was used for random-priming cDNA synthesis with SuperScript II (Invitrogen), and quantitative PCR was performed with Power SYBR Green master mix in an Applied Biosystems 7900HT Fast qRT–PCR machine. Primers used for gene expression are indicated in Supplementary Table 5. Extracts were prepared by direct suspension of cells in Laemmli buffer followed by three pulses of sonication for 15 s at 15% amplitude (Branson Digital Sonifier). SDS-PAGE and immunoblots were performed using standard methods.

### Mcm3-Lox mRNA sequencing

Total RNA from Mcm3^Lox/Lox^ MEFs was isolated and cDNA was prepared as indicated in the previous section. Three overlapping fragments covering the whole Mcm3-Lox cDNA molecule were amplified by PCR (primer sequences are shown in Supplementary Table 6) and sequenced. Experimental sequences were compared with the targeting vector used by Genoway and the NCBI reference sequence for Mcm3 mRNA (NM_008563.2). DNA and encoded protein alignments were performed with Clustal Omega (EMBL-EBI website).

### Immunofluorescence microscopy

Primary MEFs or primary erythroid populations were incubated in μCLEAR bottom polylysine-treated 96-well or 384-well plates (Greiner Bio-One) for 2 h at 37 °C, fixed in 4% paraformaldehyde in PBS for 15 min at RT and permeabilizad with 0.5% Triton-X100 in PBS (5 min at RT). Cells were incubated in blocking solution (1% bovine serum albumin in PBS) for 30 min. Primary antibody solution was applied for 1 h at RT. For RPA immunostaining, soluble proteins were extracted prior to fixation with 0.5% Triton X-100 in CSK buffer (10 mM Pipes-KOH pH 7.0, 100 mM NaCl, 300 mM sucrose, 3 mM MgCl_2_). Cell nuclei were stained with DAPI (Sigma). Images were acquired either in a Leica-TCS SP5X confocal microscope, with a HCX PL APO 20x objective using LAS AF software or in an Opera High-Content Screening System (PerkinElmer) with an APO 20 ×, 0.7 NA water-immersion objective using Acapella software (PerkinElmer). To estimate the number of RPA foci-positive cells or γH2AX foci-positive cells, 100–200 or 250–500 cells were scored respectively in each condition. To measure intensity of MCM3 or γH2AX in erythroid populations, images were acquired from each well, nuclei were marked by DAPI staining and protein immunostaining intensity was measured within the nuclei.

### Single-molecule analysis of DNA replication

Exponentially growing MEFs or single-cell suspensions of fetal livers were pulse-labeled with 50 μM CldU (20 min) followed by 250 μM IdU (20 min). Labeled cells were harvested and resuspended in 0.2 M Tris pH 7.4, 50 mM EDTA and 0.5% SDS. Stretched DNA fibers were prepared as described^52^. For immunodetection of labeled tracks, fibers were incubated with primary antibodies for 1 h at RT and the corresponding secondary antibodies for 30 min at RT, in a humidity chamber. DNA was stained with anti-ssDNA to assess fiber integrity. Fiber images were obtained in a DM6000 B Leica microscope with an HCX PL APO 40 ×, 0.75 NA objective. The conversion factor used was 1 μm=2.59 kb. In each assay, >300 individual tracks were measured for FR estimation, >100 fibers containing two or more origins were analyzed for IOD estimation, >200 labeled fibers were counted for fiber length and origin density, and >180 bidirectional forks were counted for fork symmetry.

### Histology and immunohistochemistry (IHC)

Embryos, normal tissues and tumour samples were fixed in 10% buffered formalin (Sigma) and embedded in paraffin using standard procedures. For histopathological studies, 3 μm sections were stained with haematoxylin and eosin (HE). The following antibodies were used for IHC analysis: MCM3 (generated in rabbits immunized with synthetic peptide N-CSQEDTEQKRKRRK-C conjugated to KLH; Sigma-Genosys, UK; serum was used at 1:500 dilution), Ki67 (Master Diagnostica, 0003110QD; 1:500), γH2AX (Millipore, 05-636; 1:100), pS15-p53 (Cell Signaling, 9284; 1:200), Ter119 (BD, 550565; 1:50), Pax5 (Santa Cruz Biotechnology, sc-1974; 1:50), CD3 (Santa Cruz Biotechnology, sc-1127; 1:50) and FVIII (Dako, A0082; 1:200). Tissue slides were digitalized using a Mirax scanner (Carl Zeiss) and equivalent areas per tissue and group were analyzed using AxioVision digital image processing software (Carl Zeiss). Areas of positive staining were normalized to the total analyzed area.

### Fluorescence activated cell sorting of hematopoietic cells

To prepare fetal liver cell suspensions, E14.5–16.5 fetal livers were disaggregated in 2% FBS-RPMI by multiple passages through a 26-gauge needle and filtered through a 70 μm strainer (BD Falcon). c-kit-positive cells were enriched by positive selection using streptavidin-conjugated magnetic beads and an autoMACS cell separator (Miltenyi Biotec, Auburn, CA). Depletion of Lineage (Lin)+ cells was done by incubation with purified Lin antibodies (see below) followed by magnetic beads (Dynal, Life Technologies) as per manufacturer instructions. HSCs were stained with unconjugated Lin antibodies Gr-1 (1:800), Mac1 (1:100), B220 (1:800), CD3 (1:100), CD4 (1:100), CD5 (1:100), CD8 (1:100), Ter-119 (1:100), obtained from the UCSF Hybridoma Core Facility), goat anti-rat-PE-Cy5 (Invitrogen, A10691; 1:400), c-Kit-APC-eFluor780 (eBioscience, 47-1171-82; 1:800), Sca-1-PB (BioLegend, 108120; 1:400), Flk2-Bio (eBioscience, 13-1351-82; 1:100), CD48-Alexa Fluor 647 (BioLegend, 103416; 1:400), CD150-PE (BioLegend, 115904; 1:400) and SA-PE-Cy7 (eBioscience, 25-4317-82; 1:800) antibodies. FcγR-PerCP-eFluor710 (eBioscience, 46-0161-82; 1:400) and CD34-FITC (eBioscience, 11-0341-85; 1:25) were included for concomitant isolation of MPPs and GMPs, and granulocytes were stained with Mac-1-PE-Cy7 (eBioscience, 25-0112-82; 1:3200) and Gr-1-PB (eBioscience, 57-5931-82; 1:400) antibodies. Stained cells were re-suspended for final analysis in Hanks Buffered Salt Solution (HBSS) with 2% heat-inactivated fetal calf serum (FCS) and 1 μg/ml propidium iodide (PI) for dead cell exclusion. Cell isolation was performed on a FACSAriaII (BD, San Jose, CA) and cell analysis was performed on a LSRII (BD, San Jose, CA).

E13.5-15.5 erythroid populations were characterized using Lineage-APC (1:100), Ter119-FITC (1:200) and CD71-PE (1:200) conjugated antibodies from Pharmingen (San Jose, CA). Stained cells were resuspended in PBS, 0.2% BSA, 3 mM EDTA and run in a LSR Fortessa (BD, San Jose, CA). Pulse processing was used to exclude cell aggregates and 1 μg/ml DAPI was added for dead cell exclusion. At least 20,000 events were collected from the single alive population. For DNA content analyses, fetal liver suspensions were fixed in 4% paraformaldehyde in PBS for 15 min at RT. After fixation, cells were washed in PBS and stained with 5 μg/ml Hoescht (Invitrogen). For apoptosis assays, cells were labelled with surface markers Ter119 and CD71, fixed, permeabilized and labelled with anti-activated Caspase 3 (BD Biosciences; 1:200). Cell sorting was performed on a FACSAriaII (BD, San Jose, CA) and data was analyzed with FlowJo 9.7.5 (Tree star, Ashland, OR).

### Blood sampling

After embryo exsanguination, peripheral blood was washed and analyzed for erythrocyte parameters on an Abacus Junior Vet Hematology Blood Analyzer (Diatron) or deposited onto coverslips using a Thermo-Fisher Shandon Cytospin 2 (600 r.p.m. 5 min, mounted on slides and stained with Giemsa as described^28^. To determine the percentage of nucleated cells in E14.5 embryonic blood samples, cells were stained with Ter119-FITC (1:200; Pharmingen, San Jose, CA) and nucleated/enucleated cells were differentiated with 5 μg ml^−1^ Hoescht (Invitrogen).

### Steady-state and stress erythropoiesis response

Mice with constitutive expression of fluorescent tdTomato from the *Rosa26* locus were generated by germline recombination of the *Rosa-CAG-LSL-tdTomato* allele^29^, carried by the *Ai14* reporter mouse line (stock 007914, Jackson Laboratory). Total cells were recovered from the bone marrow of CAG-Tomato mice and from the fetal livers of E14.5 Mcm3^+/+^ or Mcm3^Lox/Lox^ C57Bl/6 donor mice, and transplanted into lethally irradiated C57BL/6 primary female recipients (10^6^ cells per mouse; 5 mice per group). 8 weeks post-transplantation, donor chimerism (Tom+/Tom-) was analyzed in peripheral blood after staining with Ter119. To monitor the erythropoietic stress response, mice were subjected to phleblotomy (400 μl peripheral blood per 25 g of body weight). 4 days post-phlebotomy, accumulation of RBC progenitors EryA, EryB and EryC in the spleen was monitored as described^30^, differentiating between Tom+ and Tom- cells.

### HSC transplantation

Fetal HSCs (Lin-/Sca-1+/c-Kit+/Flk2-/CD150+/CD48-) were isolated from the fetal livers of E14.5 Mcm3^+/+^, Mcm3^+/Lox^ or Mcm3^Lox/Lox^ CD45.2 C57Bl/6 donor mice, and transplanted into lethally irradiated CD45.1 C57Bl/6 primary recipients (50 fetal HSCs per mouse; 5 mice per group) together with 300,000 Sca-1-depleted CD45.1 helper BM cells. 4 months post-transplantation, the primary recipients were sacrificed to assess the percentage of donor-derived chimerism in the bone marrow (BM), spleen, peripheral blood and HSC compartment and to re-isolate donor-derived adult MCM3^+/+^ or MCM3^Lox/Lox^ HSCs^53^. Donor chimerism was analyzed using CD45.2-FITC, (eBioscience 12-0453-83; 1:50), B220-APC-e780 (47-0452-82; 1:800), Gr-1-PB (1:400), Mac-1-PE-Cy7 (1:3200), CD3-e660 (eBioscience, 50-0032-82; 1:400) and Ter-119-PE-Cy5 (eBioscience 15-5921-83; 1:800) antibodies. Re-isolated CD45.2^+^ donor-derived adult MCM3^+/+^ or MCM3^Lox/Lox^ HSCs were transplanted into lethally irradiated CD45.1 secondary recipients (500 HSCs per mouse; 5 mice per group) together with 300,000 Sca-1-depleted CD45.1 helper BM cells.

### Single-cell tracking of division kinetics

HSCs isolated from the fetal liver of E14.5–16.5 embryos were sorted into 96-well plates (1 cell/ well) and visually inspected after 12 h to confirm successful single cell sort. At the indicated time points, cell cultures were monitored to establish the kinetics of the first cell division (appearance of 2 or more cells). 96 wells were scored per condition.

### HSC colony formation

100 HSCs were cultured in Iscove’s modified Dulbecco’s media (IMDM) supplemented with 5% FBS (StemCell Technology, 06200), 1 × penicillin/streptomycin, 0.1 mM non-essential amino acids, 1 mM sodium pyruvate, 2 mM L-glutamine and 50 μM 2-mercaptoethanol (base media) supplemented with the following cytokines (all from PeproTech): IL-3 (10 ng ml^−1^), GM-CSF (10 ng ml^−1^), SCF (25 ng ml^−1^), IL-11 (25 ng ml^−1^), Flt-3L (25 ng ml^−1^), Tpo (25 ng ml^−1^) and Epo (4 U ml^−1^) (culture media). When indicated, 50 ng ml^−1^ aphidicolin (Sigma, A4487) was added to the culture media. Colonies (triplicates) were counted on day 7. All cultures were maintained at 37 °C in a 5% CO2 water jacket incubator (Thermo Scientific).

### Statistical methods

In column graphs, data are expressed as mean±s.d. Statistical analyses were done using Fisherŕs test. When the data are presented in scatter dot plots, the bar corresponds to the median value. For the analyses of FR, IOD and fork symmetry parameters in stretched DNA fibers and immunofluorescence data from acquired in the high-throughput Opera system, data distribution was normally not Gaussian, and differences between samples were assessed with the nonparametric Mann-Whitney rank-sum test. In the analysis of apoptosis and cell death in EB populations, the mean values derived from 3 independent experiments were compared by One-Way Anova and further Bonferroni post-test. Statistical analysis was performed in Prism v4.0 (GraphPad Software).

## Additional information

**How to cite this article:** Alvarez, S. *et al*. Replication stress caused by low MCM expression limits fetal erythropoiesis and hematopoietic stem cell functionality. *Nat. Commun.* 6:8548 doi: 10.1038/ncomms9548 (2015).

## Supplementary Material

Supplementary Information Supplementary Figures 1-9 and Supplementary Tables 1-6

## Figures and Tables

**Table 1:** Origin density in Mcm3^+/++/+^ and Mcm3-deficient EBs.

	R1		R2		R3		R4	
	+/++/+	L/L	+/++/+	L/L	+/++/+	L/L	+/++/+	L/L
n. oris	327	382	325	344	285	292	285	250
DNA, Mb	141,3	118,4	110,9	108,7	89,91	84,24	85,29	69,10
ori, Mb	2,32	3,23	2,93	3,16	3,17	3,47	3,34	3,62

Proliferating EBs derived from the fetal liver of Mcm3^+/+^ or Mcm3^Lox/Lox^ (L/L) embryos were labeled with CldU and IdU and isolated at different maturation stages (R1-R4) by flow cytometry after CD71/Ter119 staining. Origin density was calculated by measuring the number of origin structures in multiple microscopy fields, until a minimum of 250 origins were scored per condition, and the total length of DNA in every fiber containing at least one replication structure.

## References

[b1] Costa A., Hood I. V. & Berger J. M. Mechanisms for initiating cellular DNA replication. Annu. Rev. Biochem. 82, 25–54 (2013). 23746253 10.1146/annurev-biochem-052610-094414 PMC4696014

[b2] Masai H., Matsumoto S., You Z., Yoshizawa-Sugata N. & Oda M. Eukaryotic chromosome DNA replication: where, when and how? Annu. Rev. Biochem. 79, 89–130 (2010). 20373915 10.1146/annurev.biochem.052308.103205

[b3] Bicknell L. S. . Mutations in ORC1, encoding the largest subunit of the origin recognition complex, cause microcephalic primordial dwarfism resembling Meier-Gorlin syndrome. Nat. Genet. 43, 350–355 (2011a). 21358633 10.1038/ng.776

[b4] Bicknell L. S. . Mutations in the pre-replication complex cause Meier-Gorlin syndrome. Nat. Genet. 43, 356–359 (2011b). 21358632 10.1038/ng.775 PMC3068194

[b5] Guernsey D. L. . Mutations in origin recognition complex gene ORC4 cause Meier–Gorlin syndrome. Nat. Genet. 43, 360–364 (2011). 21358631 10.1038/ng.777

[b6] Gineau L. . Partial MCM4 deficiency in patients with growth retardation, adrenal insufficiency and natural killer cell deficiency. J. Clin. Invest. 122, 821–832 (2012). 22354167 10.1172/JCI61014 PMC3287233

[b7] Pruitt S. C., Bailey K. J. & Freeland A. Reduced Mcm2 expression results in severe stem/progenitor cell deficiency and cancer. Stem Cells 25, 3121–3132 (2007). 17717065 10.1634/stemcells.2007-0483

[b8] Shima N. . A viable allele of Mcm4 causes chromosome instability and mammary adenocarcinomas in mice. Nat. Genet. 39, 93–98 (2007). 17143284 10.1038/ng1936

[b9] Chuang C. H., Wallace M. D., Abratte C., Southard T. & Schimenti J. C. Incremental genetic perturbations to MCM2-7 expression and subcellular distribution reveal exquisite sensitivity of mice to DNA replication stress. PLoS Genet. 6, e1001110 (2010). 20838603 10.1371/journal.pgen.1001110 PMC2936539

[b10] Kunnev D. . DNA damage response and tumorigenesis in Mcm2-deficient mice. Oncogene 29, 3630–3638 (2010). 20440269 10.1038/onc.2010.125 PMC2892019

[b11] Kawabata T. . Stalled fork rescue via dormant replication origins in unchallenged S phase promotes proper chromosome segregation and tumor suppression. Mol. Cell. 41, 543–553 (2011a). 21362550 10.1016/j.molcel.2011.02.006 PMC3062258

[b12] Kawabata T. . A reduction of licensed origins reveals strain-specific replication dynamics in mice. Mamm. Genome 22, 506–517 (2011b). 21611832 10.1007/s00335-011-9333-7 PMC3528403

[b13] Bagley B. N. . A dominantly acting murine allele of Mcm4 causes chromosomal abnormalities and promotes tumorigenesis. PLoS Genet. 8, e1003034 (2012). 23133403 10.1371/journal.pgen.1003034 PMC3486839

[b14] Williams G. H. & Stoeber K. The cell cycle and cancer. J. Pathol. 226, 352–364 (2012). 21990031 10.1002/path.3022

[b15] Tsaniras S. C. . Licensing of DNA replication, cancer, pluripotency and differentiation: an interlinked world? Semin. Cell. Dev. Biol. 30, 174–180 (2014). 24641889 10.1016/j.semcdb.2014.03.013

[b16] Blow J. J., Ge X. Q. & Jackson D. A. How dormant origins promote complete genome replication. Trends. Biochem. Sci. 36, 405–414 (2011). 21641805 10.1016/j.tibs.2011.05.002 PMC3329722

[b17] Woodward A. M. . Excess Mcm2-7 license dormant origins of replication that can be used under conditions of replicative stress. J. Cell Biol. 173, 673–683 (2006). 16754955 10.1083/jcb.200602108 PMC2063885

[b18] Ge X. Q., Jackson D. A. & Blow J. J. Dormant origins licensed by excess Mcm2-7 are required for human cells to survive replicative stress. Genes Dev. 21, 3331–3341 (2007). 18079179 10.1101/gad.457807 PMC2113033

[b19] Ibarra A., Schwob E. & Méndez J. Excess MCM proteins protect human cells from replicative stress by licensing backup origins of replication. Proc. Natl Acad Sci. USA 105, 8956–8961 (2008). 18579778 10.1073/pnas.0803978105 PMC2449346

[b20] Hyrien O., Maric C. & Mechali M. Transition in specification of embryonic metazoan DNA replication origins. Science 270, 994–997 (1995). 7481806 10.1126/science.270.5238.994

[b21] Sasaki T., Sawado T., Yamaguchi M. & Shinomiya T. Specification of regions of DNA replication initiation during embryogenesis in the 65-kilobase DNApolalpha-dE2F locus of Drosophila melanogaster. Mol. Cell. Biol. 19, 547–555 (1999). 9858578 10.1128/mcb.19.1.547 PMC83912

[b22] Gregoire D., Brodolin K. & Mechali M. HoxB domain induction silences replication origins within the locus and specifies a single origin at its boundary. EMBO. Rep. 7, 812–816 (2006). 16845368 10.1038/sj.embor.7400758 PMC1525151

[b23] Dazy S., Gandrillon O., Hyrien O. & Prioleau M. N. Broadening of DNA replication origin usage during metazoan cell differentiation. EMBO Rep. 7, 806–811 (2006). 16799461 10.1038/sj.embor.7400736 PMC1525144

[b24] Norio P. . Progressive activation of DNA replication initiation in large domains of the immunoglobulin heavy chain locus during B cell development. Mol. Cell. 20, 575–587 (2005). 16307921 10.1016/j.molcel.2005.10.029

[b25] Mechali M. Eukaryotic DNA replication origins: many choices for appropriate answers. Nat. Rev. Mol. Cell. Biol. 11, 728–738 (2010). 20861881 10.1038/nrm2976

[b26] Norio P. DNA replication: the unbearable lightness of origins. EMBO Rep. 7, 779–781 (2006). 16880822 10.1038/sj.embor.7400766 PMC1525147

[b27] Pop R. . A key commitment step in erythropoiesis is synchronized with the cell cycle clock through mutual inhibition between PU.1 and S-phase progression. PLoS Biol. 21, 8 (2010). 10.1371/journal.pbio.1000484 PMC2943437 20877475

[b28] Fraser S. T., Isern J. & Baron M. H. Maturation and enucleation of primitive erythroblasts during mouse embryogenesis is accompanied by changes in cell-surface antigen expression. Blood 109, 343–352 (2007). 16940424 10.1182/blood-2006-03-006569 PMC1785074

[b29] Madisen L. . A robust and high-throughput Cre reporting and characterization system for the whole mouse brain. Nat. Neurosci. 13, 133–140 (2010). 20023653 10.1038/nn.2467 PMC2840225

[b30] Liu Y. . Suppression of Fas-FasL coexpression by erythropoietin mediates erythroblast expansion during the erythropoietic stress response in vivo. Blood 108, 123–133 (2006). 16527892 10.1182/blood-2005-11-4458 PMC1895827

[b31] Lopez-Contreras A. J., Gutierrez-Martinez P., Specks J., Rodrigo-Perez S. & Fernandez-Capetillo O. An extra allele of Chk1 limits oncogene-induced replicative stress and promotes transformation. J. Exp. Med. 209, 455–461 (2012). 22370720 10.1084/jem.20112147 PMC3302228

[b32] Flach J. . Replication Stress is a Potent Driver of Functional Decline in Aging Hematopoietic Stem Cells. Nature 512, 198–202 (2014). 25079315 10.1038/nature13619 PMC4456040

[b33] Wang J. . A differentiation checkpoint limits hematopoietic stem cell self-renewal in response to DNA damage. Cell 148, 1001–1014 (2012). 22385964 10.1016/j.cell.2012.01.040

[b34] Alver R. C., Chadha G. S. & Blow J. J. The contribution of dormant origins to genome stability: from cell biology to human genetics. DNA. Repair. (Amst). 19, 182–189 (2014). 24767947 10.1016/j.dnarep.2014.03.012 PMC4065331

[b35] Kozar K. . Mouse development and cell proliferation in the absence of D-cyclins. Cell 118, 477–491 (2004). 15315760 10.1016/j.cell.2004.07.025

[b36] Kinross K. M., Clark A. J., Iazzolino R. M. & Humbert P. O. E2f4 regulates fetal erythropoiesis through the promotion of cellular proliferation. Blood 108, 886–895 (2006). 16861343 10.1182/blood-2005-09-008656

[b37] Sankaran V. G., Orkin S. H. & Walkley C. R. Rb intrinsically promotes erythropoiesis by coupling cell cycle exit with mitochondrial biogenesis. Genes Dev. 22, 463–475 (2008). 18258751 10.1101/gad.1627208 PMC2238668

[b38] Hu T. . Concomitant inactivation of Rb and E2f8 in hematopoietic stem cells synergizes to induce severe anemia. Blood 119, 4532–4542 (2012). 22422820 10.1182/blood-2011-10-388231 PMC3362366

[b39] Chapman J. R. . RIF1 is essential for 53BP1-dependent nonhomologous end joining and suppression of DNA double-strand break resection. Mol. Cell 49, 858–871 (2013). 23333305 10.1016/j.molcel.2013.01.002 PMC3594748

[b40] Monturus E. . DNA replication fading as proliferating cells advance in their commitment to terminal differentiation. Sci. Rep. 2, 279 (2012). 22359734 10.1038/srep00279 PMC3283920

[b41] Shearstone J. R. . Global DNA demethylation during mouse erythropoiesis in vivo. Science 334, 799–802 (2011). 22076376 10.1126/science.1207306 PMC3230325

[b42] Conti C. . Replication fork velocities at adjacent replication origins are coordinately modified during DNA replication in human cells. Mol. Biol. Cell 18, 3059–3067 (2007). 17522385 10.1091/mbc.E06-08-0689 PMC1949372

[b43] Pietras E. M., Warr M. R. & Passegué E. Cell cycle regulation in hematopoietic stem cells. J. Cell Biol. 195, 709–720 (2011). 22123859 10.1083/jcb.201102131 PMC3257565

[b44] Rossi D. J. . Cell intrinsic alterations underlie hematopoietic stem cell aging. Proc. Natl Acad Sci. USA 102, 9194–9199 (2005). 15967997 10.1073/pnas.0503280102 PMC1153718

[b45] Rossi D. J. . Deficiencies in DNA damage repair limit the function of haematopoietic stem cells with age. Nature 447, 725–729 (2007). 17554309 10.1038/nature05862

[b46] Chambers S. M. . Aging hematopoietic stem cells decline in function and exhibit epigenetic dysregulation. PLoS Biol. 5, e201 (2007). 17676974 10.1371/journal.pbio.0050201 PMC1925137

[b47] Mohrin M. . Hematopoietic stem cell quiescence promotes error-prone DNA repair and mutagenesis. Cell Stem Cell 7, 174–185 (2010). 20619762 10.1016/j.stem.2010.06.014 PMC2924905

[b48] Beerman I., Seita J., Inlay M. A., Weissman I. L. & Rossi D. J. Quiescent Hematopoietic Stem Cells Accumulate DNA Damage during Aging that Is Repaired upon Entry into Cell Cycle. Cell Stem Cell 15, 37–50 (2014). 24813857 10.1016/j.stem.2014.04.016 PMC4082747

[b49] Rossi D. J., Jamieson C. H. & Weissman I. L. Stems cells and the pathways to aging and cancer. Cell 132, 681–696 (2008). 18295583 10.1016/j.cell.2008.01.036

[b50] Geiger H., de Haan G. & Florian M. C. The ageing haematopoietic stem cell compartment. Nat. Rev. Immunol. 13, 376–389 (2013). 23584423 10.1038/nri3433

[b51] Festing M. F. Design and statistical methods in studies using animal models of development. ILAR. J. 47, 5–14 (2006). 16391426 10.1093/ilar.47.1.5

[b52] Mourón S. . Repriming of DNA synthesis at stalled replication forks by human PrimPol. Nat. Struct. Mol. Biol. 20, 1383–1389 (2013). 24240614 10.1038/nsmb.2719

[b53] Warr M. R. . FOXO3A directs a protective autophagy program in haematopoietic stem cells. Nature 494, 323–327 (2013). 23389440 10.1038/nature11895 PMC3579002

