# Supplementary material for: Replication stress caused by low MCM expression limits fetal erythropoiesis and hematopoietic stem cell functionality
Source: Nat Commun. 2015 Oct 12;6:8548. doi: 10.1038/ncomms9548 (PMC4608254; doi:10.1038/ncomms9548)
Supplement: Supplementary Information — Supplementary Figures 1-9 and Supplementary Tables 1-6 [file ncomms9548-s1.pdf]

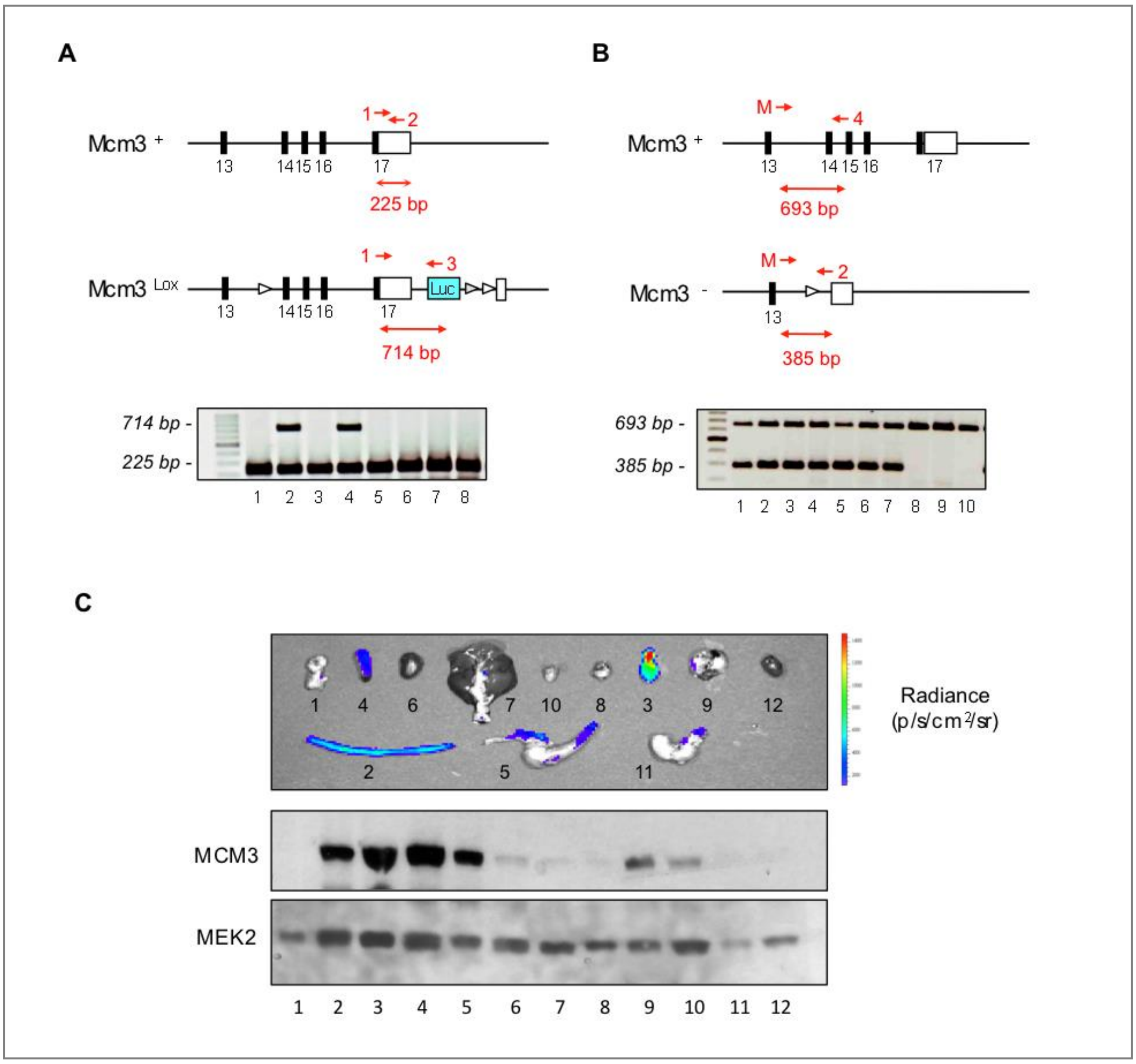

Supplementary Figure 1. Genotyping strategies for Mcm3$^{+/+}$, Mcm3$^{+/Lox}$ and Mcm3$^{+/-}$ mice and luciferase activity in Mcm3$^{+/Lox}$ mice. A. Upper part, three-primer PCR strategy at the Mcm3 locus yielding a 225 bp product in the wild-type allele and a 714 bp product in the Mcm3-*Lox* allele. Arrows indicate primer positions. Bottom part, genotyping examples of eight mice, of which #2 and #4 are Mcm3$^{+/Lox}$. **B.** Upper part, three-primer strategy yielding a 693 bp product in the wild-type Mcm3 allele and a 385 bp product in the Mcm3-null allele. Bottom part, genotyping examples of 10 mice, of which #1 to #7 were Mcm3$^{+/-}$. **C.** Top, bioluminescent signal in internal organs of Mcm3$^{+/Lox}$ mice. Bottom, MCM3 protein level determined by immunoblot in extracts prepared from the same tissues. MEK2 levels are shown as reference. Full immunoblots are shown in Supplementary Fig. 9B. Tissue code: 1, pancreas; 2, small intestine; 3, testis; 4, spleen; 5, large intestine; 6, kidney; 7, liver; 8, seminal vesicle; 9, lung; 10, bladder; 11, stomach; 12, heart.

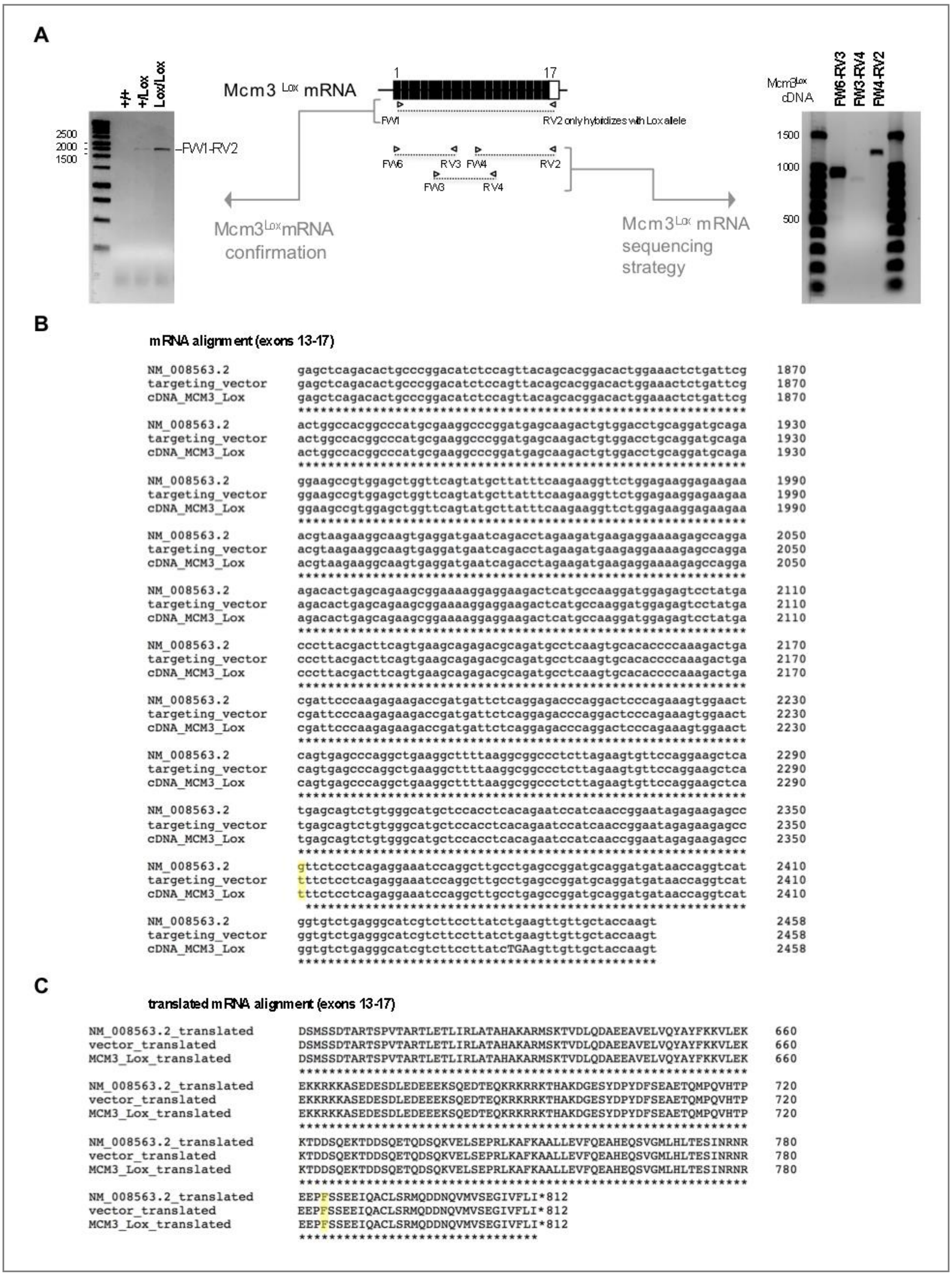

Supplementary Figure 2. Complete mRNA sequencing of the Mcm3-Lox allele. A. Schematic representation of the Mcm3-Lox mRNA. Left, PCR reactions confirming the presence of Mcm3-Lox mRNA only in MEFs of Mcm3$^{+/Lox}$ and Mcm3$^{Lox/Lox}$ genotype. Right, PCR reactions showing amplification of three overlapping cDNA fragments used for sequencing. **B**. Alignment of the experimental mRNA-derived Mcm3-Lox sequence with the murine Mcm3 NCBI reference sequence (NM_008563.2) and the targeting vector used for mouse generation. Only the last four exons are shown for clarity. Numbers are positions from ATG (+1). The experimental sequence was identical

(100%) to that of the targeting vector. One silent point mutation (G-T) at position 2351 (highlighted in yellow) was found in both the experimental and vector sequences, relative to the reference sequence. The corresponding amino acid in MCM3 protein (Phe784) remained invariant. **C**. Alignment of the corresponding protein sequences. Numbers indicate positions from the +1 Met amino acid.

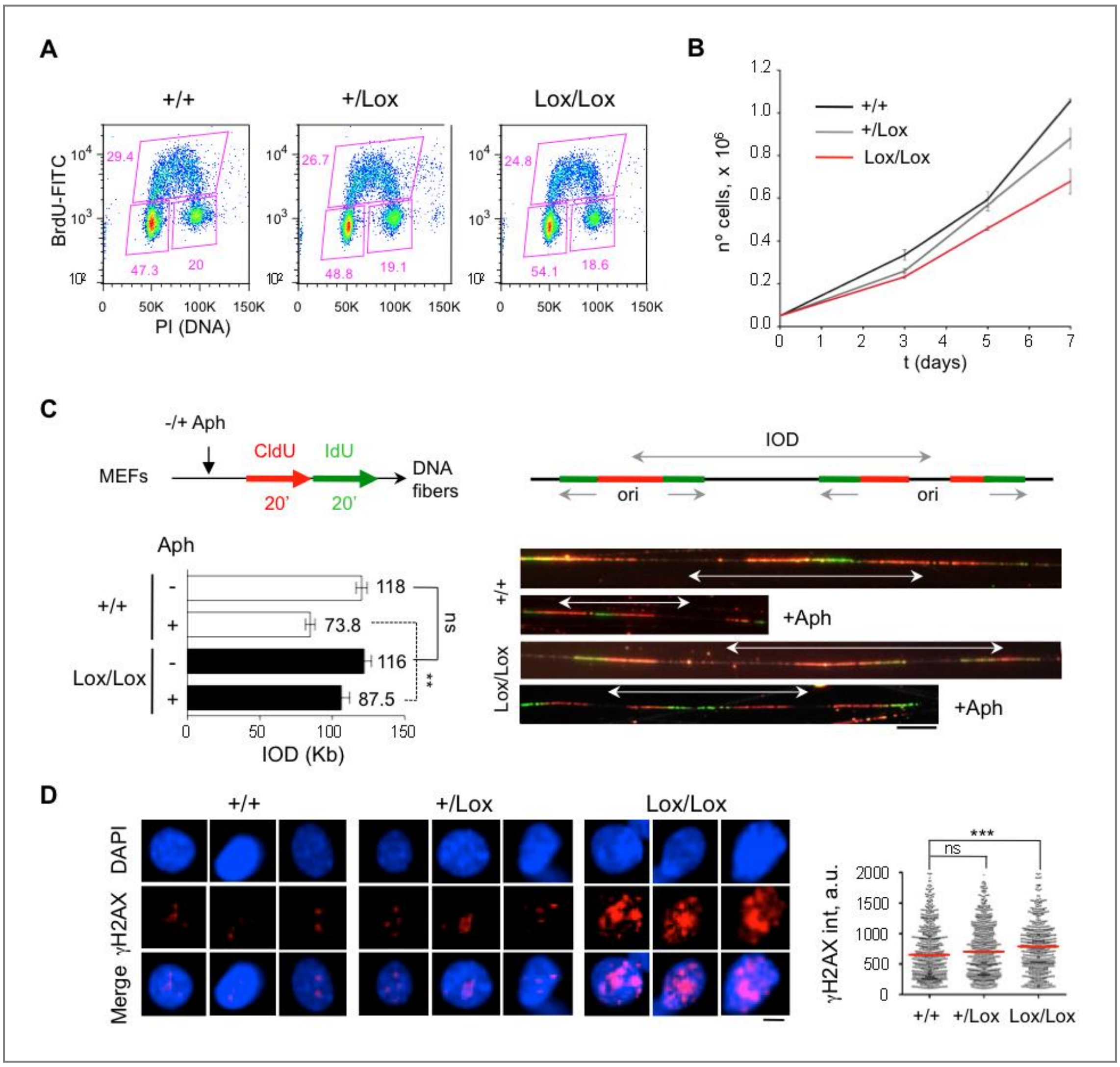

Supplementary Figure 3. DNA replication with reduced activation of dormant origins in Mcm3$^{Lox/Lox}$ MEFs. A. BrdU incorporation profiles in Mcm3$^{+/+}$, Mcm3$^{+/Lox}$ and Mcm3$^{Lox/Lox}$ MEFs. The percentage of cells in the G1, S and G2/M phases of the cell cycle are indicated. **B.** Proliferation curves of Mcm3$^{+/+}$, Mcm3$^{+/Lox}$ and Mcm3$^{Lox/Lox}$ MEFs. Error bars represent SD of duplicates. **C.** Top left, outline of the experiment. MEFs were sequentially labeled with CldU (red) and IdU (green), in the absence or presence of 0.5 µM aphidicolin. Top right, schematic of a DNA molecule with two active origins, representing the fibers used to measure inter-origin distance (IOD). Bottom left, quantification of IOD of Mcm3$^{+/+}$, Mcm3$^{+/Lox}$ and Mcm3$^{Lox/Lox}$ MEFs. Median IOD values are indicated (70-100 IODs scored for each condition). Experiments were done in triplicate and statistical analysis was done with Fisher's test (** p<0.01; n.s. not significant). Bottom right, fiber examples. Scale bar, 10 µm. **D.** Confocal microscopy images of γH2AX nuclear staining in Mcm3$^{+/+}$, Mcm3$^{+/Lox}$ and Mcm3$^{Lox/Lox}$ MEFs. Scale bar, 2.5 µm. Right, quantification of γH2AX nuclear intensity. Red lines indicate the median value (>700 nuclei scored per condition). Data are representative of 3 independent experiments. P-values were calculated by Mann-Whitney test (*** p<0.001; ns, not significant).

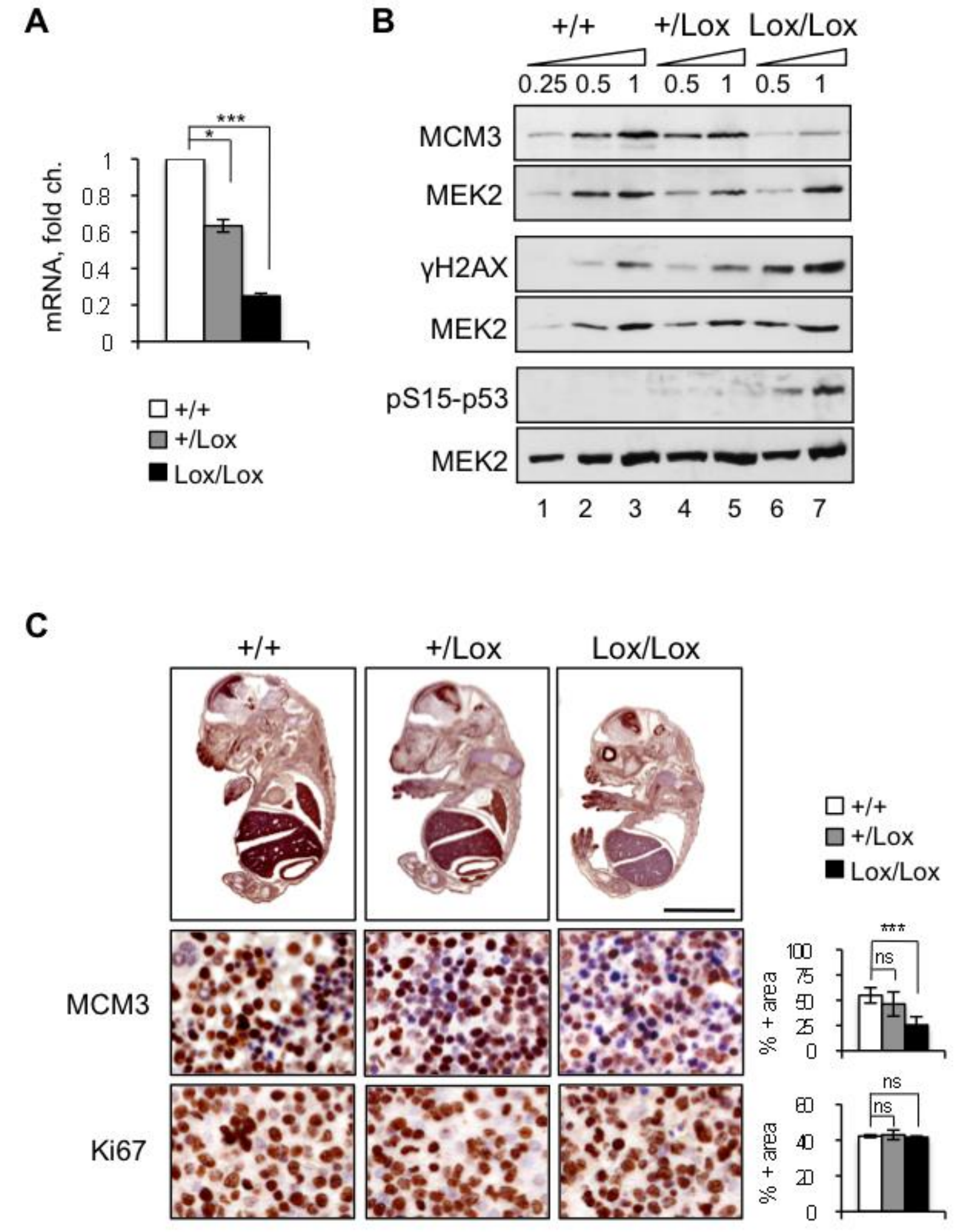

Supplementary Figure 4. Low expression of Mcm3 in the fetal liver of Mcm3$^{Lox/Lox}$ embryos. A. Mcm3 mRNA expression in the fetal liver of Mcm3$^{+/+}$, Mcm3$^{+/Lox}$ and Mcm3$^{Lox/Lox}$ embryos, as determined by qRT-PCR. Histogram bars represent average value ± SD of 3 independent experiments. **B.** Immunoblots showing total MCM3, γH2AX and pS15-p53 protein levels in fetal liver extracts. For accurate comparisons, different amounts of each extract were loaded. In each SDS-PAGE, MEK2 levels are shown as loading control. Full immunoblots are shown in Supplementary Fig. 9C. **C.** Top, IHC detection of MCM3 protein in Mcm3$^{+/+}$, Mcm3$^{+/Lox}$ and Mcm3$^{Lox/Lox}$ whole-embryo (E16.5) sections. Scale bar, 5 mm. Lower panels, IHC of MCM3 and Ki67 and γH2AX in the fetal liver. Scale bar, 25 μM. Signal quantifications represents staining-positive area (average value ± SD) in the fetal liver of Mcm3$^{+/+}$ (n=4 for MCM3, n=2 for Ki67); Mcm3$^{+/Lox}$ (n=5 for MCM3, n=2 for Ki67) and Mcm3$^{Lox/Lox}$ (n=5 for MCM3, n=2 for Ki67) embryos. P-values were calculated by Fisher's test (***p<0.001; n.s. not significant).

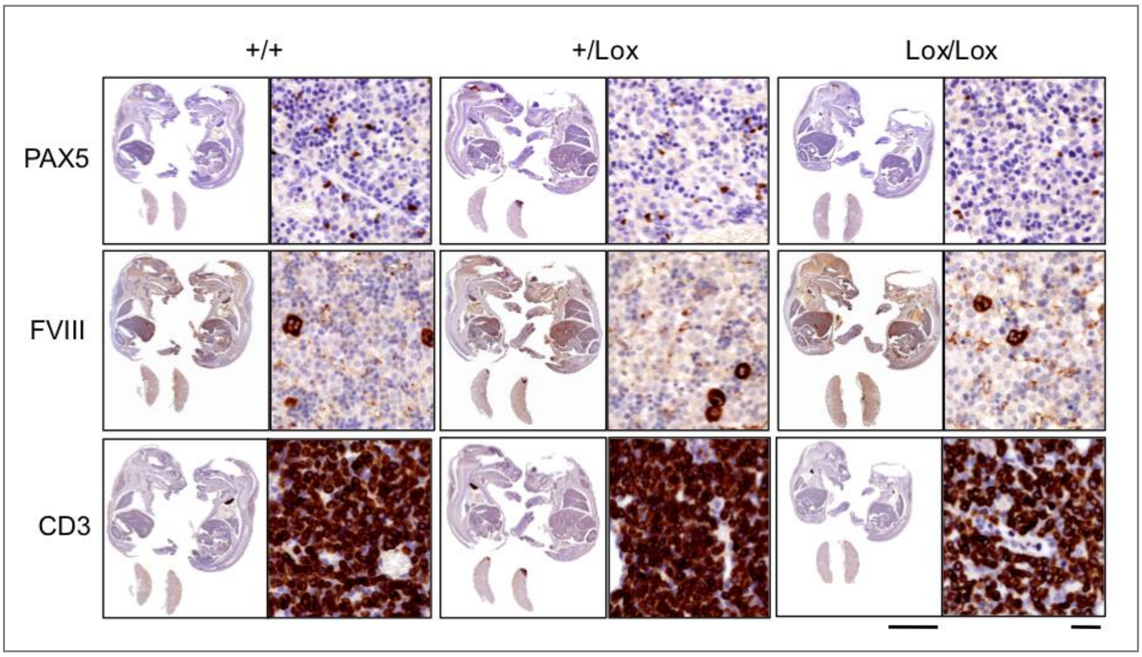

Supplementary Figure 5. IHC stainings of mature hematopoietic populations in fetal liver and thymus. IHC stainings of B-lymphocytes (Pax5), megakaryocytes (FVIII) and T-lymphocytes (CD3) in MCM3$^{+/+}$, MCM3$^{+/Lox}$ and MCM3$^{Lox/Lox}$ whole embryos**.** Scale bar, 5 mm. Magnifications show Pax5 and FVIII staining in the fetal liver and CD3 staining in the fetal thymus. Scale bar, 50 μm.

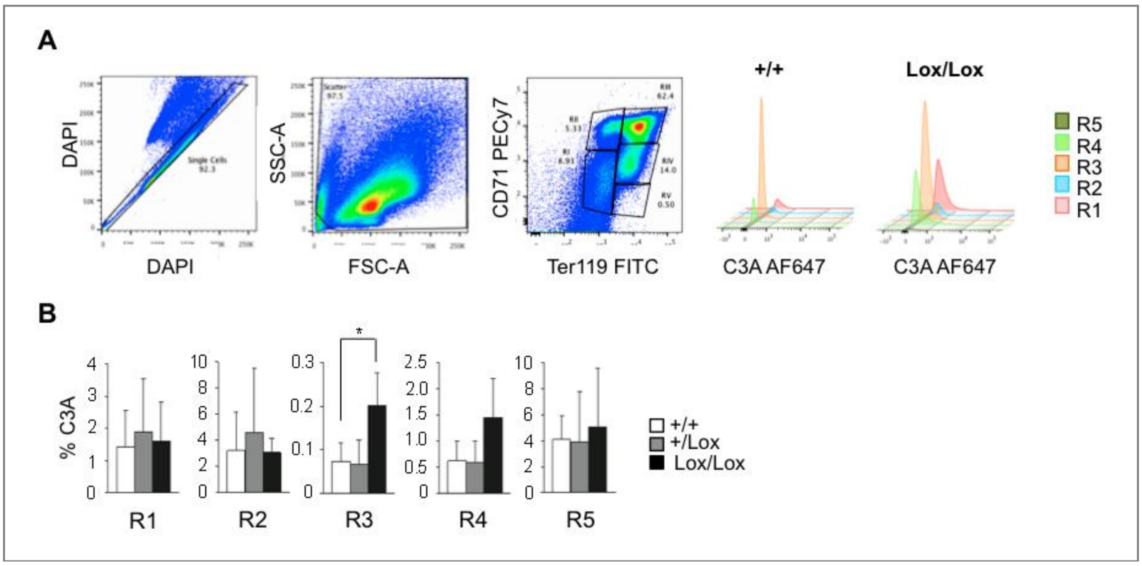

Supplementary Figure 6. Detection of activated caspase 3 in Mcm3$^{Lox/Lox}$ EBs. A. Examples of cytometric detection of activated caspase 3 (C3A) in R1-R5 EBs previously gated using CD71/Ter119. See Materials and Methods for details. **B.** Quantification of the percentage of C3A-positive cells in each population (n=5 Mcm3$^{+/+}$, n=6 Mcm3$^{+/Lox}$, n=6 Mcm3$^{Lox/Lox}$. * denotes $p < 0.05$ in One-way Anova test).

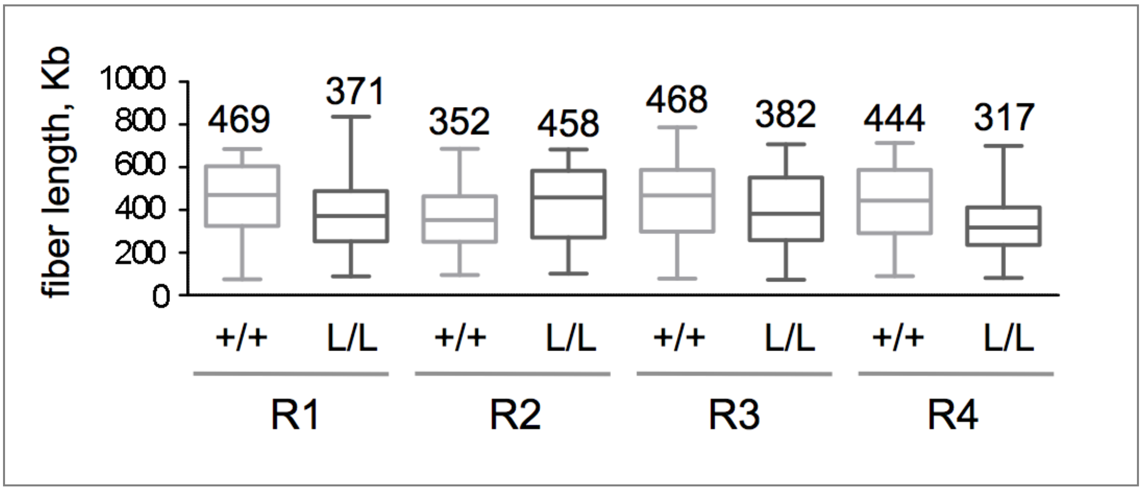

Supplementary Figure 7. Quantification of DNA fiber length in the experiments shown in Main Figure 5. Box-plot representation of DNA fiber lengths. A minimum of 200 fibers labeled with at least one replication structure (origin/fork/termination) were counted in each condition. The median value of fiber length is indicated in each case.

A

+/Lox : CHK1$^{+/Tg}$ x +/Lox : CHK1$^{+/Tg}$

	**Lox/Lox : CHK1$^{+/+}$**	**Lox/Lox :CHK1$^{+/Tg}$**
obtained	**2.8%**	**7.6%**
expected	12.5%	12.5%

B

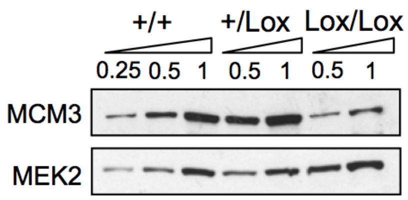

Supplementary Figure 8. Partial rescue of Mcm3$^{Lox/Lox}$ embryonic lethality by Chk1 overexpression. A. Percentages of mice of the indicated genotypes (obtained/expected) derived from continued breeding of Mcm3$^{+/Lox}$: Chk1$^{+/Tg}$ mice. **B.** Immunoblots showing MCM3 protein levels in whole fetal liver extracts of Mcm3$^{+/+}$, Mcm3$^{+/Lox}$, Mcm3$^{Lox/Lox}$ embryos in the Chk1$^{+/Tg}$ genetic background. The amount of MCM3 protein in Mcm3$^{Lox/Lox}$ extracts is comparable to that of the C57BL/6 background, shown in Supplementary Fig. 4. Full immunoblots are shown in Supplementary Fig. 9D.

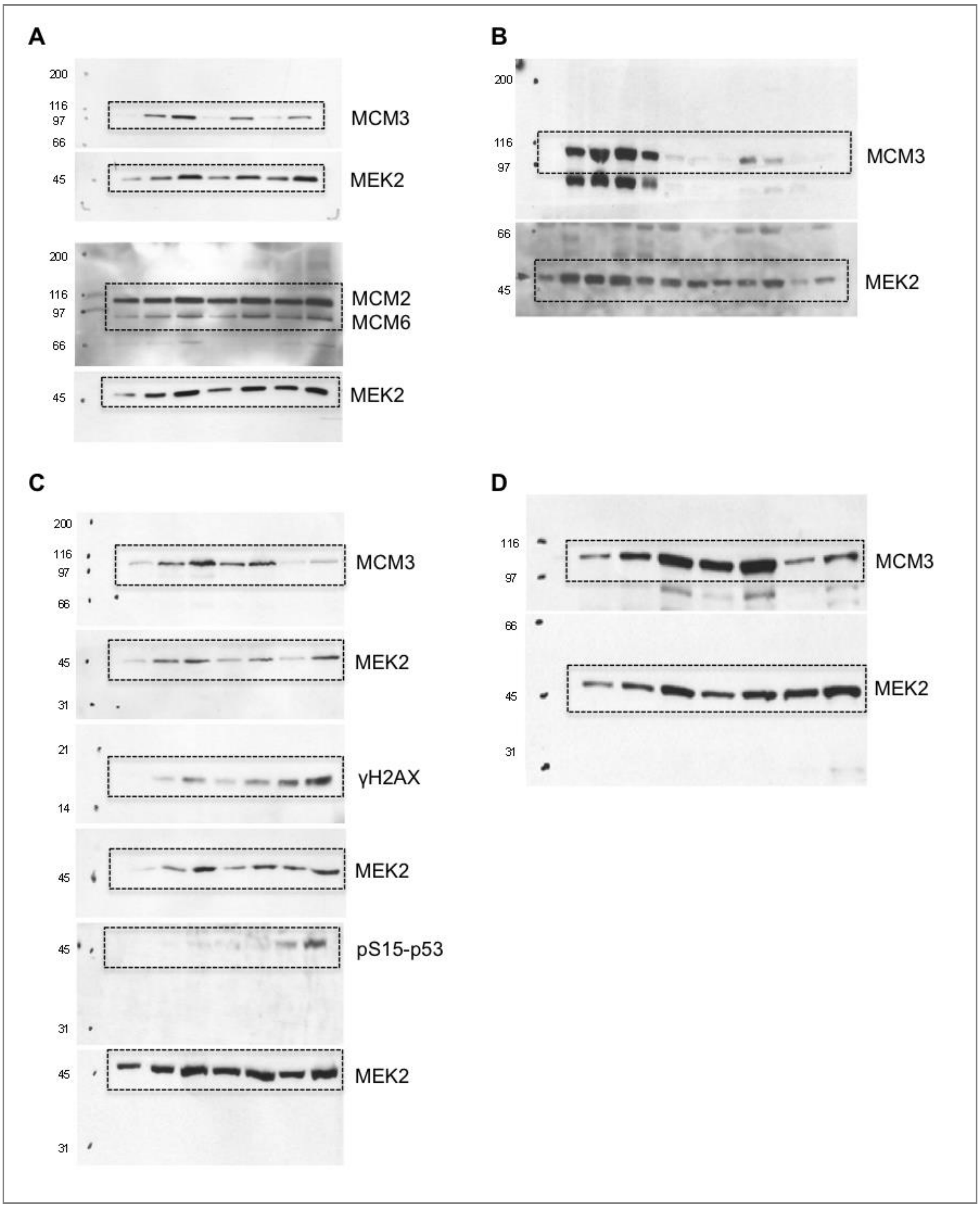

Supplementary Figure 9. Full immunoblots. This figure includes larger areas of the immunoblots corresponding to (**A**) Figure 1F; (**B**) Supplementary Figure 1C; (**C**) Supplementary Figure 4B; (**D**) Supplementary Figure 8B. Dashed boxes correspond to the area shown in the main figures.

Case	Age (months)	Diagnosis	Location	IHC
BBG107	20	Lymphoma	Spleen	H&E
BBG115	30,5	Lymphoma	Liver	H&E
			Lymphatic gland	H&E
			Spleen	H&E
			Kidney	H&E
		Adenomatous hyperplasia	Thyroid	H&E
BBG132	29	Follicular lymphoma	Spleen	H&E
		Infiltration	Lung	H&E
		Follicular adenoma	Thyroid	H&E
		Epithelial hyperplasia	Skin	H&E
		Polyp	Intestine	H&E
BBG185	28	Histiocytic sarcoma	Spleen	H&E
BBG193	27,5	Lymphoma<br>Lymphoma infiltration	Spleen<br>Adipose tissue	H&E<br>H&E
BBG602	18	Adenocarcinoma	Harderian gland	H&E
BBG621	18,5	Bronchioalveolar adenocarcinoma (II)	Lung	H&E
		Hyperplasia	Forestomach	H&E
BBG656	19	Lymphoma	Spleen	H&E
			Peripancreatic gland	H&E

Supplementary Table 1. Summary of tumor incidence in $MCM3^{+/+}$ mice.

Case	Age (months)	Diagnosis	Location	IHC
BBG51*	30	Adenocarcinoma	Lung	H&E
		Hepatocarcinoma	Liver	H&E
BBG52*	30	Histiocytic sarcoma	Skin	H&E
			Stomach	H&E
			Lung	H&E
			Lymphatic gland	H&E
BBG54*	33	Histiocytic sarcoma	Liver	H&E
BBG55*	33	Histiocytic sarcoma	Liver	F4/80
BBG60*	28	Lymphoma	Mesenteric glanglion	H&E
			Kidney	H&E
			Spleen	PAX5/ CD3/ F4/80
BBG66*	22	Lymphoma	Spleen	H&E
BBG137*	23	Histiocytic sarcoma	Liver	CD3/ F4/80
BBG145*	26	Lymphoma	Mesenteric glanglion	H&E
			Spleen	H&E
			Adipose tissue	H&E
BBG150*	22	Adenocarcinoma	Mammary gland	H&E
BBG170*	28	Angiosarcoma	Spleen	H&E
BBG177*	23	Lymphoma	Lymphatic glands	H&E
			Mesenteric glanglion	H&E
			Ureter	H&E
			Thymus	H&E
			Lung	H&E
			Bone marrow	H&E
BBG181*	25	Lymphoma	Spleen	PAX5/ CD3
			Liver	H&E
			Mesenteric ganglion	H&E
			Kidney	H&E
BBG190*	25	Lymphoma	Spleen	H&E
		Adenocarcinoma	Lung	H&E
BBG202*	21	Papilloma	Stomach	H&E
BBG203*	21	Lymphoma	Spleen	H&E

			Mesenteric ganglion	H&E
			Pancreas	H&E
			Lung	H&E
BBG235*	28	Lymphoma	Spleen	H&E
			Adipose tissue	H&E
			Mammary gland	H&E
		Hepatocarcinoma	Liver	H&E
		Adenoma	Small intestine	H&E
			Thyroid	H&E
BBG261*	20	Lymphoma	Spleen	PAX5
BBG295*	20	Lymphoma	Mesenteric ganglion	PAX5/ CD3
			Spleen	H&E
			Kidney	H&E
			Lung	H&E
			Mammary gland	H&E
		Lipoma	Adipose tissue	H&E
BBG335	23	Follicular hyperplasia	Thyroid	H&E
BBG340*	20	Angiosarcoma	Bone	CD31
			Adipose tissue	H&E
			Spleen	H&E
BBG427	21.5	Lymphoma	Spleen	H&E
			Adipose tissue	H&E
			Lymphatic glands	H&E
			Pancreas	H&E
		Papilloma	Intestine	H&E
			Stomach	H&E
		Adenomatous hyperplasia	Thyroid	H&E
		Cystic hyperplasia	Endometrium	H&E
BBG518	23	Lymphoma	Spleen	H&E
			Bone marrow	H&E
			Prostate	H&E
			Epididyme	H&E
BBG632	21	Lymphoma	Spleen	H&E
			Bone marrow	H&E
		Follicular hyperplasia	Thyroid	H&E
BBG664	12	Leiomyosarcoma	Adipose tissue	Actin /p-p53
			Skin	

		Histiocytic sarcoma	Muscle	F4/80 /CD3
BBG802	12	Adenoma	Thyroid	H&E
		Lipoma	Adipose tissue	H&E

Supplementary Table 2. Summary of tumor incidence in $MCM3^{+/Lox}$ and $MCM3^{+/Lox\text{-}Neo}$ (*) mice.

Case	Age (months)	Diagnosis	Location	IHC
BBG111	30,5	Lymphoma	Lymphatic glands	H&E
			Liver	H&E
			Spleen	H&E
		Adrenal hyperplasia	Kidney	H&E
		Papilloma	Stomach	H&E
		Serous cystadenome	Uterus	H&E
		Adenomatous hyperplasia	Small intestine	H&E
BBG264	21	Lymphoma	Peripancreatic gland	CD3/ PAX5/ F4/80
			Kidney	H&E
			Liver	H&E
			Lung	H&E
		Follicular hyperplasia	Thyroid gland	H&E
		Prostate hyperplasia	Prostate	H&E
BBG269	23	Adenocarcinoma	Lung	H&E
BBG271	23	Lymphoma	Pancreas	CD3/ PAX5
		Follicular adenoma	Spleen, mesenteric and thoracic ganglions	CD3/ PAX5
		Ductal hyperplasia	Mammary gland	H&E
BBG275	21	Adenocarcinoma	Lung	H&E
		Hyperplasia	Thyroid	H&E
BBG277	23	Mesothelioma	Mesenteric ganglion	H&E
		Hyperplasia	Thymus	H&E
BBG464	17,5	Lymphoma	Peripancreatic gland	H&E
BBG624	17	Hepatocarcinoma	Liver	H&E
		Adenoma	Small intestine	H&E
BBG640	13	Histiocytic sarcoma (B-cell type)	Liver	H&E
			Adipose tissue	H&E
			Spleen	H&E
			Lung	H&E
			Lymphatic ganglions	H&E
			Testis	H&E
			Bladder	H&E
BBG642	18	Lymphoma	Intestine	H&E

			Liver	H&E
		Adrenal hyperplasia	Kidney	H&E
		Hyperplasia	Bladder	H&E
		Myeloid hyperplasia	Bone Marrow	H&E
BBG644	12	Adenoma	Thyroid/ Parathyroid	H&E
		Hyperplasia (Leydig cells)	Testis	H&E
BBG651	12	Lymphoma	Spleen	Pax5
			Lymphatic glands	H&E
			Adipose tissue	H&E
		Cholangioma	Liver	Cytokeratin 19/ CD31
BBG689	17,5	Lymphoma	Kidney	H&E
		Follicular hyperplasia	Thyroid	H&E
BBG943	15,5	Hyperplasia	Stomach	H&E

Supplementary Table 3. Summary of tumor incidence in $MCM3^{+/-}$ mice.

Allele	Primer name	Sequence (5'→3')
Mcm3-Lox	1	GTGTCTGAGGGCATCGTCTT
	2	CCTCACAAGCCCAATCCTAA
	3	GCAAGCTGACCCTGAAGTTC
Mcm3-null	M	TGCCTATGGCTCATCTGAAGAACTGC
	2	CCTCACAAGCCCAATCCTAA
	4	TCTCCCAGTGTTCTTGAGGC

Supplementary Table 4. Oligonucleotide primers used for mice genotyping**.**

Gene	Primer	Sequence (5'→3')
Mcm3	*MCM3*-Fw	TTCCTCAGCTGTGTGGTCTG
	MCM3-Rv	TCACCACCCTAGTGGCTTTC
Gapdh	GAPDH-Fw	TGAAGCAGGCATCTGAGGG
	GAPDH-Rv	CGAAGGTGGAAGAGTGGGAG

Supplementary Table 5. Oligonucleotide primers used for qRT-PCR.

Gene	Primer	Sequence (5'→3')
Mcm3	Fw1	ATTTCGGAGCTGCGAC
	Fw3	CCATCACCATCCAGGAGATG
	Fw4	AAGCTGGTGCTATGGTCCTG
	Fw6	CGTTTCCGGACTGTTTGGT
	Rv2	CGATTCTTAAGTGATACTTGGTAGC
	Rv3	AGCAGAGGATTGCCTTCTTG
	Rv4	GAACTACCCAATGGCAAAGC

Supplementary Table 6. Oligonucleotide primers for used for DNA sequencing.
